# Short-term and bystander effects of radiation on murine submandibular glands

**DOI:** 10.1242/dmm.049570

**Published:** 2022-11-10

**Authors:** Hitoshi Uchida, Matthew H. Ingalls, Eri O. Maruyama, Carl J. Johnston, Eric Hernady, Roberta C. Faustoferri, Catherine E. Ovitt

**Affiliations:** ^1^ Center for Oral Biology, University of Rochester Medical Center, Rochester, NY 14642, USA; ^2^ Department of Pediatrics, University of Rochester Medical Center, Rochester, NY 14642, USA; ^3^ Department of Radiation Oncology, University of Rochester Medical Center, Rochester, NY 14642, USA; ^4^ Department of Biomedical Genetics, University of Rochester Medical Center, Rochester, NY 14642 USA

**Keywords:** Salivary glands, Radiation, Small-animal radiation research platform, Bystander effects

## Abstract

Many patients treated for head and neck cancers experience salivary gland hypofunction due to radiation damage. Understanding the mechanisms of cellular damage induced by radiation treatment is important in order to design methods of radioprotection. In addition, it is crucial to recognize the indirect effects of irradiation and the systemic responses that may alter saliva secretion. In this study, radiation was delivered to murine submandibular glands (SMGs) bilaterally, using a ^137^Cs gamma ray irradiator, or unilaterally, using a small-animal radiation research platform (SARRP). Analysis at 3, 24 and 48 h showed dynamic changes in mRNA and protein expression in SMGs irradiated bilaterally. Unilateral irradiation using the SARRP caused similar changes in the irradiated SMGs, as well as significant off-target, bystander effects in the non-irradiated contralateral SMGs.

## Teaser

**Summary:** Rapid changes in murine gene expression occur in irradiated salivary glands within hours of irradiation, and in non-irradiated contralateral glands, due to bystander effects.

## INTRODUCTION

Over 800,000 new cases of head and neck cancers are diagnosed annually in the world and are treated with a combination of radiation (IR), chemotherapy and surgery (Cramer et al., 2019). A consequence of radiotherapy for head and neck cancer is hyposalivation, manifested as a significant reduction in saliva flow and permanent loss of the secretory acinar cells (Pinna et al., 2015). Hyposalivation can cause burning mouth, dental caries, gingivitis, periodontitis and oral infections, as well as difficulty in speaking, chewing and swallowing, reducing quality of life. Available treatments are only temporary and palliative (Vissink et al., 2010; Villa et al., 2015).

The slow turnover of salivary gland acinar cells is inconsistent with their acute sensitivity to IR (Vissink et al., 2015). The response of salivary glands to IR has been divided into two stages (Coppes et al., 2001; Jasmer et al., 2020). The first stage includes short-term effects that occur within hours to days, such as acute reduction in salivation, changes in saliva composition, interstitial edema and enlarged acinar cells (Coppes et al., 2001). The second stage involves long-term and irreversible effects manifested after weeks to months, including acinar cell loss, fibrosis, continued hyposalivation and absence of cell renewal (Coppes et al., 2001; Konings et al., 2005). The molecular mechanisms driving these changes are not well understood. In addition to direct effects, IR also induces responses in non-irradiated tissues, known as bystander effects (Blyth and Sykes, 2011; Daguenet et al., 2020). In minipigs, the irradiated, and non-irradiated, contralateral (CL) salivary glands showed a coupled response (Lombaert et al., 2020), but bystander effects in salivary glands have not yet been carefully investigated.

In this study, we examined the molecular mechanisms underlying the acute loss of secretory function that occurs in the first 48 h after IR. We utilized a ^137^Cs gamma ray (Cs) irradiator, with anatomical targeting (achieved using a slit collimator set-up) to deliver IR bilaterally to murine submandibular glands (SMGs) (Fig. S1A). We also employed the use of a small-animal X-irradiator [small-animal radiation research platform (SARRP)] for targeted, unilateral radiation to a single SMG (Fig. S1B-F). The SARRP uses computed tomography (CT)-image guidance to ensure precision for unilateral SMG IR and, therefore, is a more clinically relevant methodology. Results from our analyses of mRNA and protein expression indicated that similar, time-dependent changes were observed in SMGs following either unilateral (SARRP) or bilateral IR. Furthermore, we demonstrate that even without direct IR exposure, unilateral IR resulted in significant, off-target, bystander effects in the non-irradiated, CL SMG.

## RESULTS

### Short- and long-term changes in saliva secretion following IR

To trace the rate of secretory loss in murine salivary glands following IR, we measured saliva flow at 3, 24 and 48 h, and weekly for up to 12 weeks (Fig. S1G). The baseline saliva flow was established for each mouse on 3 consecutive days and was determined to be 8.59±1.80 mg/g body weight (Fig. S1H,I; *n*=11-13). Half of the mice received an IR dose of 15 Gy simultaneously to both left and right SMGs using a Cs irradiator with a slit collimator. At 3 and 48 h, secretion levels from IR-treated mice were significantly lower than baseline measurements (Fig. S1I). At 24 h, saliva flow returned almost to baseline levels in both IR-treated and non-IR-treated groups, and then decreased again in both groups by 48 h. A similar recovery was observed in all mice between the 48-h and 2-week collection points. The pattern of transient recovery at 24 h and 1 week suggests that repeated administration of ketamine affects secretion loss.

IR-treated mice showed a progressive decrease in saliva secretion between 3 and 9 weeks, which leveled off by 12 weeks (Fig. S1I). In contrast, saliva volume from non-IR-treated mice did not show significant changes over the 12-week timeline (Fig. S1H). These data provide a comprehensive picture of the IR-induced decrease in saliva secretion over the course of 12 weeks, valuable information for the development and timing of intervention strategies to prevent salivary gland hypofunction.

### IR perturbs expression of tight junction proteins

**Fig. 1.**
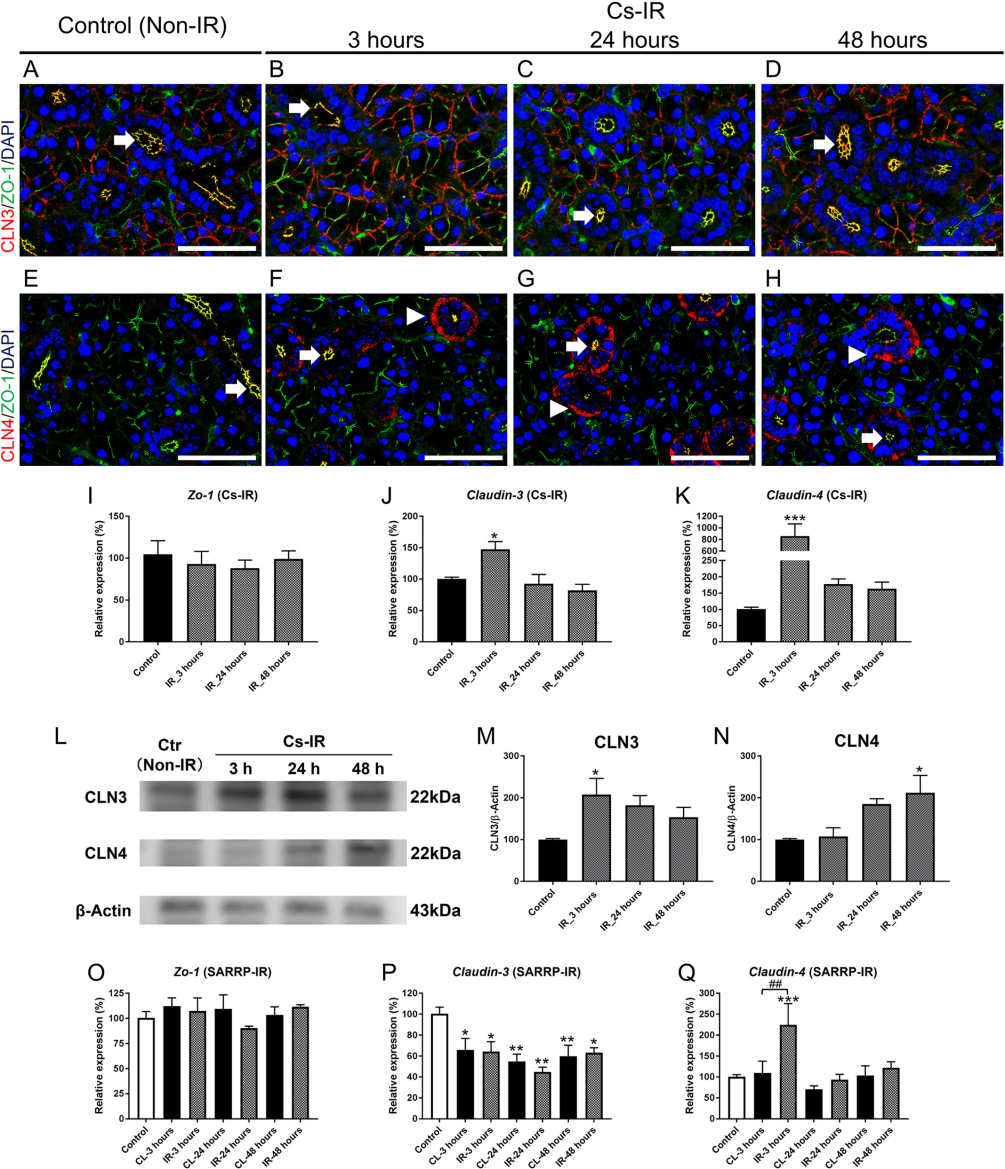
**Radiation (IR) alters expression of tight junction proteins.** (A-D) Immunofluorescent staining for ZO-1 (green) and CLN3 (red) shows expression on acinar cell membranes, and colocalization at the luminal surface of duct cells (arrows) in control (A) and irradiated submandibular glands (SMGs) at 3 h (B), 24 h (C) and 48 h (D) post-IR. (E-H) Immunohistochemistry for ZO-1 (green) and CLN4 (red) shows colocalization at the duct cell luminal surface (arrows) in control (E) and increased expression of CLN4 (arrowheads) at the basal surface of duct cells in irradiated SMGs at 3 h (F), 24 h (G) and 48 h (H) post-IR. Scale bars: 50μm. (I) Expression level of *Zo-1* mRNA did not change after IR from the ^137^Cs gamma (Cs) source (*P*>0.05 versus control for all time points). (J,K) Claudin-3 (J) and claudin-4 (K) mRNAs were upregulated at 3 h post-IR [claudin-3: *P=0.018 (3 h); claudin-4: ***P<0.001 (3 h)]. (L) Western blots to detect CLN3, CLN4 and β-actin proteins in control and irradiated SMGs using the Cs source (*n*=3). (M,N) Quantification of CLN3 and CLN4 proteins detected on western blots [CLN-3: *P=0.037 (3 h); CLN4: *P=0.027 (48 h)]. (O) *Zo-1* mRNA expression did not change in irradiated or contralateral (CL) SMGs following unilateral IR using the small-animal radiation research platform (SARRP) (one-way ANOVA; *F*=0.758; two-way ANOVA; *F*=0.728, *P*>0.05). (P) In contrast, claudin-3 mRNA was downregulated in IR and CL SMGs up to 48 h post-IR. (Q) Claudin-4 mRNA was significantly increased in irradiated compared to control (***P<0.001), but not in CL SMGs at 3 h post-IR (^##^*P*<0.01). Error bars indicate mean±s.e.m. Statistical analysis was performed compared to control (non-irradiated) using one-way ANOVA with Dunnett's post-hoc test, or comparing irradiated and CL SMGs, using two-way ANOVA with Bonferroni test (*n*=3-5 each group).

At early time points following IR, changes in cell size and interstitial space consistent with edema were observed in sections of SMGs (Fig. S2). We examined whether these changes correlated with altered expression of epithelial barrier proteins, including the tight junction proteins ZO-1 (also known as TJP1), claudin-3 (CLN3; also known as CLDN3) and claudin-4 (CLN4; also known as CLDN4). In murine SMGs, CLN3 is expressed by acinar cells, whereas CLN4 is predominantly localized to duct cells (Baker, 2016; Zhang et al., 2018). There was no significant change in ZO-1 immunostaining (Fig. 1A-H) or *Zo-1* mRNA expression levels (Fig. 1I) at 3, 24 or 48 h post-IR in mice irradiated with the Cs source. CLN3 protein staining was not altered (Fig. 1A-D); however, an increase in CLN4 staining was observed (Fig. 1F-H, arrowheads) at the basal surface of duct cells, compared to that in the control salivary glands (Fig. 1E, arrow), which expressed CLN4 only at the apical surface. A significant increase in both claudin-3 and claudin-4 mRNA was detected at 3 h post-IR (Fig. 1J,K). Consistent with previous findings (Yokoyama et al., 2017), CLN3 and CLN4 proteins showed increased expression on western blots (Fig. 1L-N).

After unilateral IR using the SARRP, *Zo-1* and claudin-4 mRNA expression patterns were similar to those in bilaterally irradiated SMGs (Fig. 1O,Q). Claudin-4 mRNA was elevated in the IR-treated SMGs, but not in the non-treated CL SMGs (Fig. 1Q). However, in contrast to the upregulation seen with the Cs source (Fig. 1J), claudin-3 mRNA expression was reduced in both SARRP-irradiated and CL SMGs (Fig. 1P). These results indicate that cell–cell junctions are perturbed both directly and indirectly after IR.

### IR transiently downregulates expression of genes required for saliva secretion

**Fig. 2.**
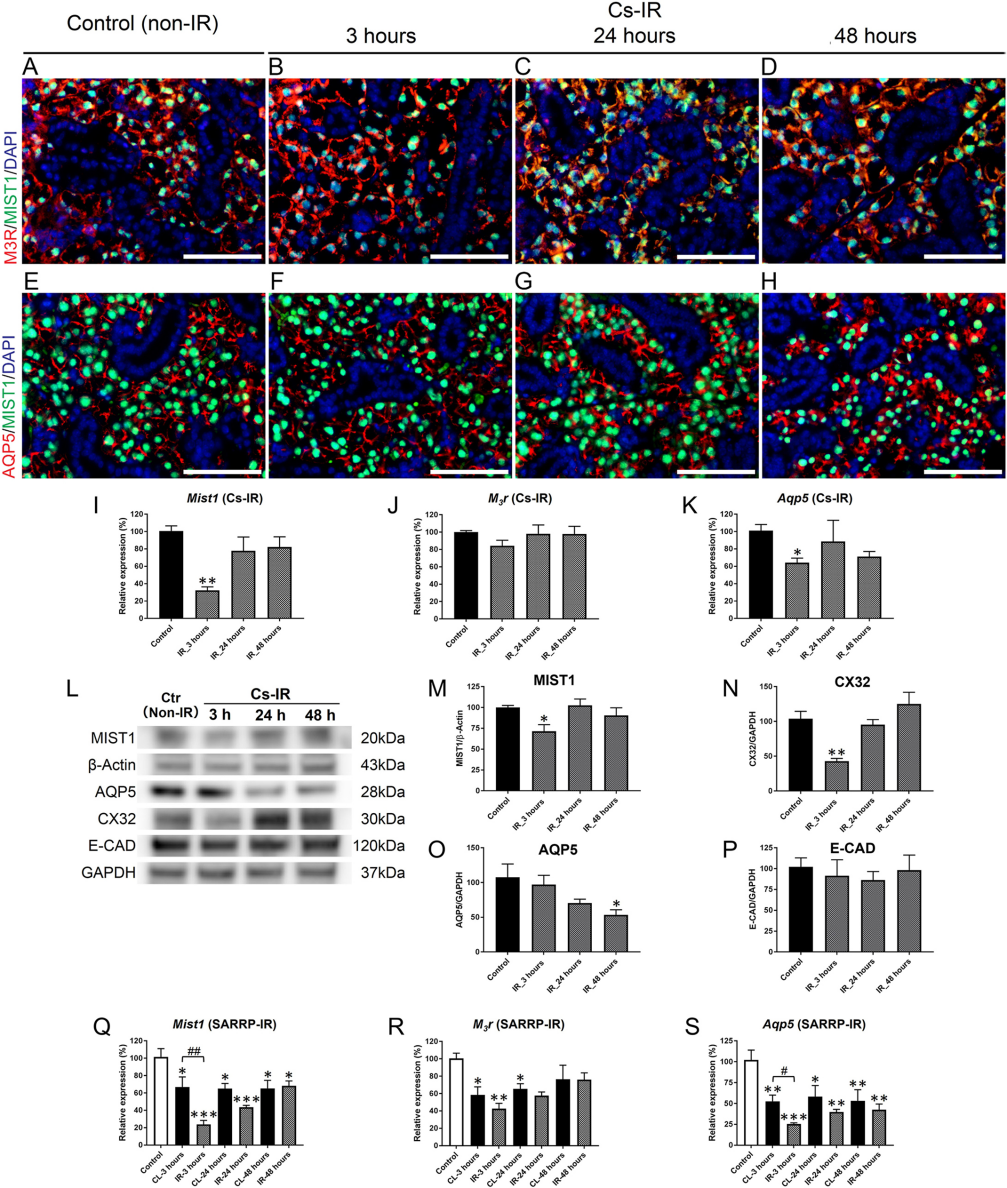
**IR transiently downregulates expression of genes involved in saliva secretion.** (A-D) Immunofluorescent staining shows colocalization of M3R^+^ (red) with MIST1^+^ (green) acinar cells (A) on control. M3R expression does not change at 3 h (B), 24 h (C) or 48 h (D) after IR using the Cs source. (E-H) AQP5 (red) localization at the apical surface of MIST1^+^ (green) acinar cells in control (E) does not change at 3 h (F), 24 h (G) or 48 h (H) post-IR. Scale bars: 100μm. (I) Expression of *Mist1* mRNA was significantly reduced at 3 h post-IR (**P=0.002), but recovered by 24 h post-IR (*P*>0.1). (J) Relative expression of *M3r* mRNA did not change post-IR using the Cs irradiator (*P*>0.1). (K) *Aqp5* mRNA expression was decreased at 3 h post-IR (*P=0.023), but recovered by 24 h post-IR (*P*>0.05). (L) Western blot of MIST1, AQP5, CX32, E-CAD, GAPDH and β-actin proteins in control and irradiated SMGs using the Cs source (*n*=3). (M,N) Densitometric analysis of MIST1 (M) and CX32 (N) MIST1: *P=0.041; CX32: **P=0.003. (O) Densitometric analysis of AQP5 protein (*P<0.05). (P) Densitometric analysis of E-CAD protein (one-way ANOVA; *F*=0.215, *P*=0.883). (Q) Following IR using SARRP, relative expression of *Mist1* mRNA was significantly decreased in both irradiated and CL SMGs at 3 h (***P<0.001, *P<0.05, respectively), at 24 h (***P<0.001, *P<0.05, respectively) and at 48 h (*P<0.05) versus control. *Mist1* expression was significantly lower in the IR-treated SMGs compared to the CL SMGs (^##^*P*<0.01). (R) IR using SARRP also decreased the relative expression of *M3r* mRNA in both irradiated and CL SMGs at 3 h (**P<0.001, *P<0.01, respectively) versus non-irradiated control. (S) Following IR using SARRP, relative expression of *Aqp5* mRNA was decreased in both irradiated and CL SMGs versus control at 3 h (***P<0.001, **P<0.01, respectively), 24 h (**P<0.01, *P<0.05, respectively) and 48 h (**P<0.01, **P<0.01, respectively). *Aqp5* expression was significantly lower in the IR-treated SMG compared to the CL SMG (^#^*P*<0.05). Error bars indicate mean±s.e.m. Statistical analysis was performed compared to control (non-irradiated) using one-way ANOVA with Dunnett's post-hoc test, or comparing irradiated and CL SMGs using two-way ANOVA with Bonferroni test (*n*=3-5 each group).

To determine whether IR disrupts proteins involved in saliva secretion, we investigated the expression profiles and localization of the water channel aquaporin 5 (AQP5) and the muscarinic receptor type 3 (M3R; also known as CHRM3), which transmits signals from the parasympathetic nervous system. M3R and MIST1 (also known as BHLHA15), an acinar cell-specific transcription factor (Pin et al., 2000), are colocalized in acinar cells. Immunostaining showed no detectable change in MIST1, M3R or AQP5 (Choi et al., 2009) protein staining at 3, 24 or 48 h after IR (Fig. 2A-H). However, *Mist1* mRNA expression was significantly decreased by 3 h post-IR and recovered by 24 h (Fig. 2I). There was no change in *M3r* mRNA levels (Fig. 2J), but *Aqp5* mRNA expression was downregulated in irradiated SMGs at 3 h post-IR (Fig. 2K). Like *Mist1*, *Aqp5* mRNA expression was recovered by 24 h (Fig. 2K). Western blots showed decreases in MIST1 (Fig. 2L,M) and connexin 32 (CX32; the gap junction protein also known as GJB1) (Fig. 2L,N) protein expression at 3 h post-IR, but both were recovered by 24 h. AQP5 protein levels were decreased by 48 h post-IR (Fig. 2L,O), consistent with an earlier report (Choi et al., 2009). As a control, we quantified E-cadherin (E-CAD; also known as CDH1), a cell adhesion protein, and found that the relative amount did not change within 48 h post-IR (Fig. 2L,P), as previously reported (Wong et al., 2018). The transient decrease in MIST1, CX32 and AQP5 expression at 3 h post-IR correlates with the initial drop in saliva secretion.

The expression profiles of *Mist1*, *M3r* and *Aqp5* mRNA responded similarly in SARRP-irradiated SMGs (Fig. 2Q-S). Interestingly, expression of these mRNAs also decreased in the CL SMGs, in comparison to non-IR-treated controls, indicating that gene expression is perturbed through both direct and indirect bystander effects.

### IR induces DNA damage and pro- and anti-apoptotic markers

X-irradiation induces the formation of DNA-damage repair foci, which include γH2AX. To assess the level of DNA damage following Cs IR, SMG sections were co-stained for γH2AX, which labels IR-induced double-stranded DNA breaks (Löbrich et al., 2010), and for the sodium/potassium/two-chloride channel (NKCC1; also known as SLC12A2), an acinar cell marker. The number of γH2AX foci was significantly increased in both acinar and duct cells at 3 h following IR (Fig. S3A,B), consistent with previous reports (Meyer et al., 2017). At 24 and 48 h post-IR, the number of γH2AX foci was lower and, notably, predominantly localized to duct cells (Fig. S3C,D), as previously observed (Marmary et al., 2016; Varghese et al., 2018). IR using SARRP also induced a high number of γH2AX foci by 3 h, which then decreased in irradiated SMGs by 48 h (Fig. S3E-H). Quantification of these data confirmed that γH2AX foci were more prevalent in duct cells than in acinar cells at all times following IR, indicating an elevated level of DNA damage in this cell population (Fig. S3I). Consistent with their DNA repair activity, mRNA expression levels of *Tgfb1*, *Foxo3a* (also known as *Foxo3*) and *Gadd45a* were rapidly, but transiently, increased by 3 h post-IR (Fig. S4A-C).

**Fig. 3.**
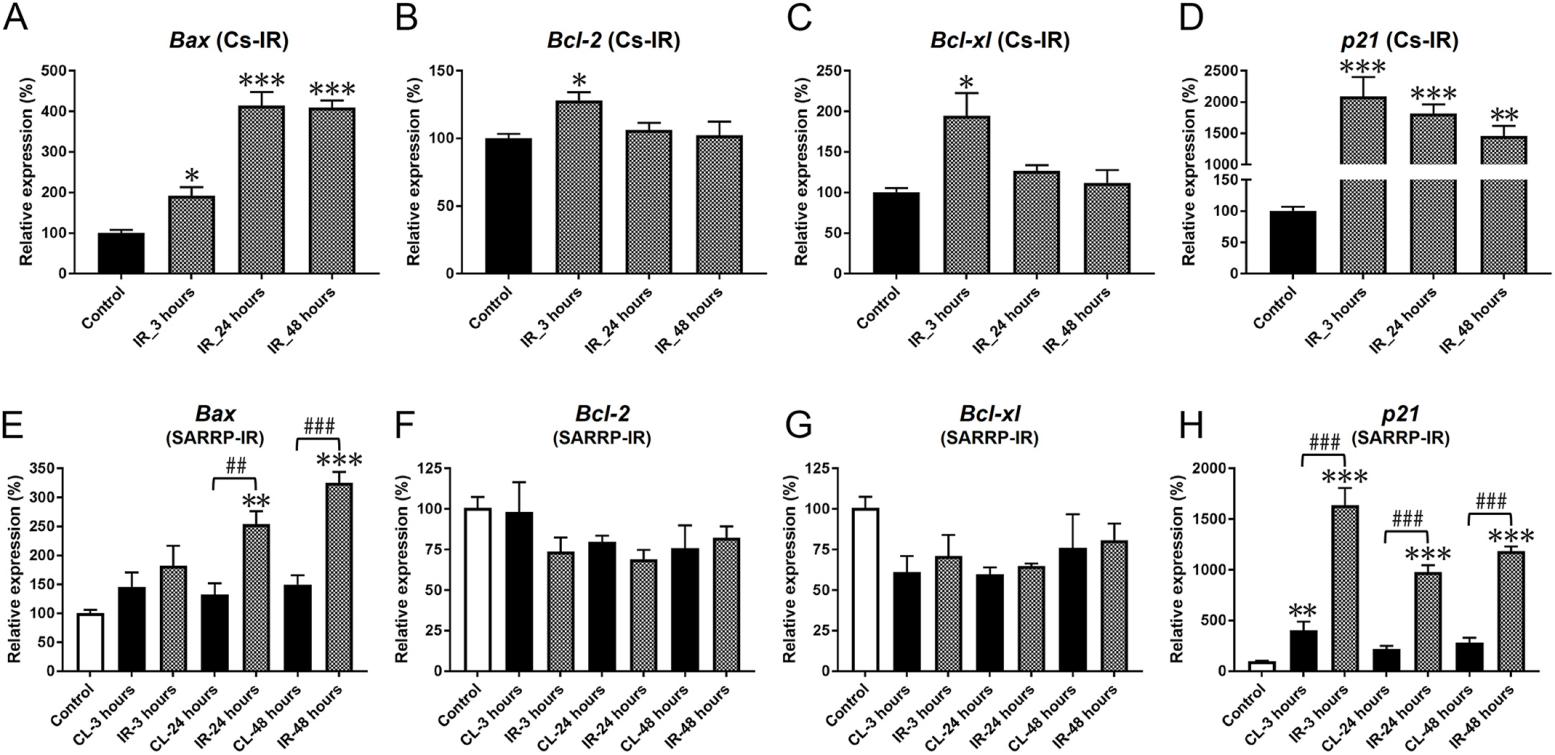
**Pro- and anti-apoptotic gene expression is induced by IR.** (A) *Bax* mRNA expression was significantly elevated at 3 h (*P=0.022), 24 h (***P<0.001) and 48 h (***P<0.001) after IR using the Cs source compared to the non-irradiated control. (B,C) mRNA expression of the anti-apoptotic factors *Bcl2* and *Bcl-xl* was transiently elevated by 3 h (*P=0.024, *P=0.003, respectively). (D) Expression of *p21* mRNA was significantly increased by 3 h (***P<0.001), and remained elevated at 24 (***P<0.001) and 48 h (**P=0.001). (E-H) After IR using SARRP, expression profiles of *Bax*, *Bcl2*, *Bcl-xl* and *p21* mRNA were similar to those observed using the Cs source. (E) *Bax* mRNA levels were elevated at 24 h (**P=0.002) and 48 h (***P<0.001), and were significantly higher in IR-treated SMGs compared to CL SMGs (^##^*P*<0.01, ^###^*P*<0.001, respectively). (F,G) Expression levels of *Bcl2* (F) and *Bcl-xl* (G) mRNA did not change significantly following IR using SARRP. (H) *p21* mRNA expression was significantly elevated at 3 h in IR-treated and CL SMGs (**P=0.005, ***P<0.001, respectively, versus control), and expression remained elevated in the IR-treated SMG up to 48 h (***P<0.001 versus control). *p21* mRNA levels in the IR-treated SMGs were significantly higher than in the CL SMGs (^###^*P*<0.001). Error bars indicate mean±s.e.m. Statistical analysis was performed compared to control (non-irradiated) using one-way ANOVA with Dunnett's post-hoc test, and to compare IR and CL SMGs using two-way ANOVA with Bonferroni test (*n*=3-5 each group).

IR of the salivary glands leads to activation of p53 (also known as TP53) (Limesand et al., 2006), which can result in cell cycle arrest or apoptosis (Riley et al., 2008). A previous study reported that the p53-dependent pro-apoptotic factors *Bax* and *PUMA* (also known as *Bbc3*) were upregulated at 4 and 8 h post-IR (Avila et al., 2009). In agreement, our data showed that *Bax* mRNA was significantly upregulated at 3, 24 and 48 h after IR using the Cs source (Fig. 3A). However, previous histological analysis of irradiated rat and mouse SMGs within 48 h post-IR did not reveal detectable apoptosis (Choi et al., 2009; Marmary et al., 2016).

To further investigate this, we examined additional targets of p53, including the anti-apoptotic factors *Bcl2*, *Bcl-xl* (also known as *Bcl2l1*) and *p21* (also known as *Cdkn1*a). mRNA expression levels of *Bcl2* and *Bcl-xl* showed transient elevation by 3 h post-IR, but returned to control levels by 24 h (Fig. 3B,C). Expression of *p21* was also rapidly and significantly upregulated by 3 h post-IR, and remained high through 48 h (Fig. 3D). The expression profiles of *Bcl2* and *Bcl-xl* were similar after IR using SARRP (Fig. 3E-H). With the exception of *p21*, there was no significant effect of IR on expression of these genes in the CL SMGs.

### Pro-inflammatory factors are rapidly upregulated following IR

**Fig. 4.**
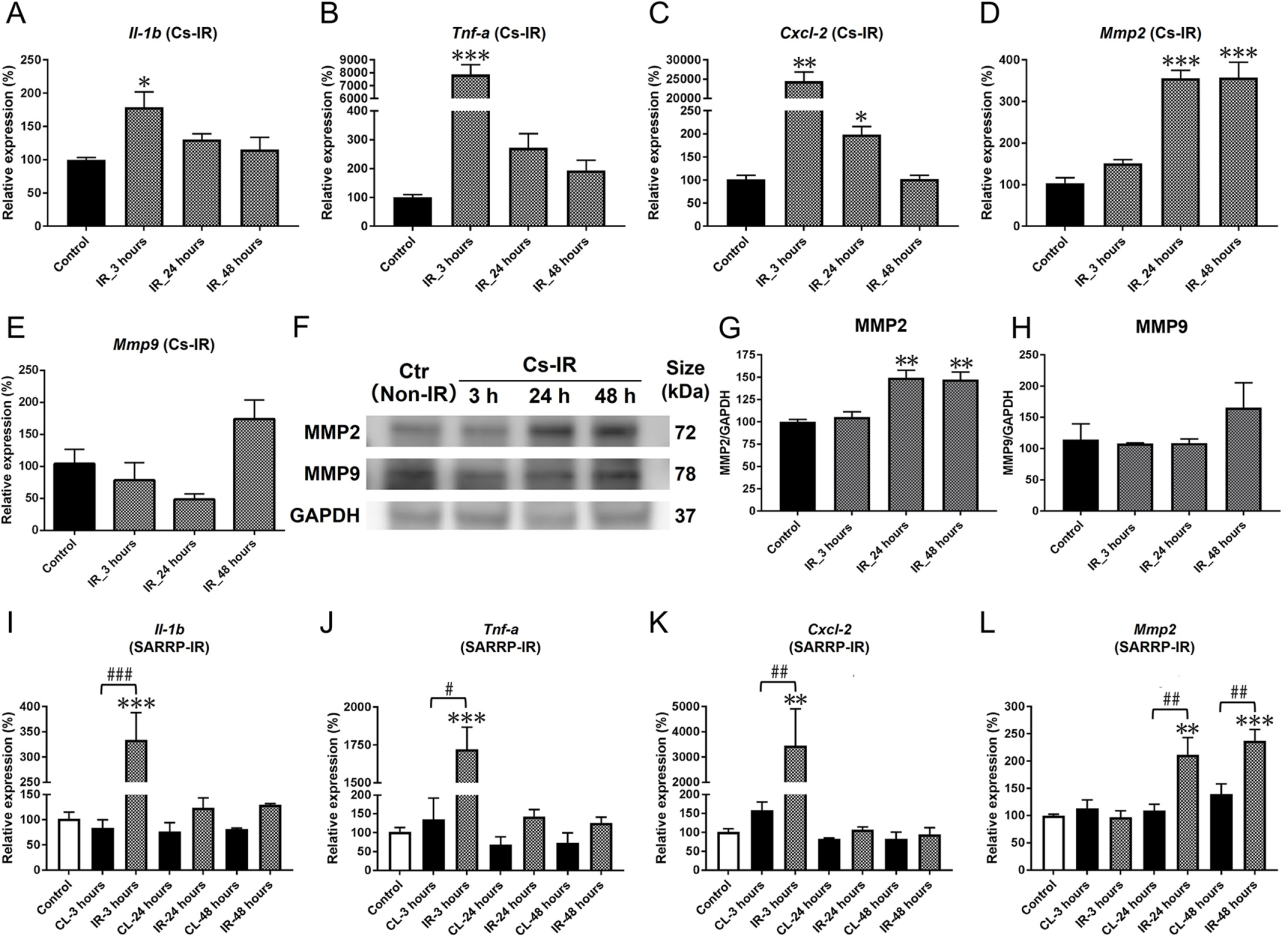
**Pro-inflammatory factors are rapidly upregulated following IR treatment.** (A,B) Expression of *Il1b* (A) and *Tnf-α* (B) mRNAs was upregulated by 3 h post-IR using the Cs source (*P=0.026, ***P<0.001, respectively). At 24 h, expression of both cytokines had decreased compared to non-irradiated control. (C) *Cxcl2* mRNA was significantly increased at 3 h (**P=0.002) and 24 h (*P=0.013) post-IR, but had decreased to control levels by 48 h post-IR. (D) Expression of *Mmp2* mRNA was increased at 24 and 48 h post-IR (***P<0.001), compared to non-irradiated control. (E) Western blot analysis of MMP2, MMP9 and GAPDH protein expression in control and irradiated SMGs (*n*=3). (F) Densitometric analysis of MMP2 protein (**P=0.002). (G) Densitometric analysis of MMP9 protein (*P*>0.1). (H-K) Following IR using SARRP, expression profiles for *Il1b* (H; ***P<0.001 versus control), *Tnf-α* (I; ***P<0.001 versus control), *Cxcl2* (J; **P=0.015 versus control) and *Mmp2* (K; **P=0.002, ***P<0.001 versus control) were similar to those induced using the Cs source. mRNA levels in IR-treated SMGs were significantly higher than in CL SMGs (^#^*P*<0.05, ^##^*P*<0.01, ^###^*P*<0.001). Error bars indicate mean±s.e.m. Statistical analysis compared to control was performed using one-way ANOVA with Dunnett's post-hoc test. Two-way ANOVA with Bonferroni test was used to compare irradiated and CL SMGs (*n*=3-5 each group)

The expression levels of pro-inflammatory cytokines were assessed in SMGs irradiated with the Cs source, using quantitative real-time PCR (qPCR). Expression of interleukin-1β (*Il1b*) and tumor necrosis factor-α (*Tnf-α*; also known as *Tnf*) mRNAs were significantly increased at 3 h post-IR (Fig. 4A,B). mRNA expression of *Cxcl2*, a pro-inflammatory cytokine produced by macrophages and found to be upregulated in oral tissues after IR (Shen et al., 2018), was also significantly increased at 3 and 24 h post-IR (Fig. 4C). Expression of *P2y2* (also known as *P2ry2*), a member of the purinergic receptor gene family shown to be upregulated by Il-1β in salivary glands after injury (Turner et al., 1997; Khalafalla et al., 2020), was also increased (Fig. S4D).

Matrix metalloproteases, such as MMP2 and MMP9, are involved in extracellular matrix tissue remodeling, a process linked to inflammation (Duarte et al., 2015), and are stimulated by IR in some cell types (Wang et al., 2000; Lombaert et al., 2020). In bilaterally irradiated SMGs, *Mmp2* mRNA expression was significantly increased at 24 and 48 h post-IR (Fig. 4D), and MMP2 protein expression was increased on western blots, relative to control protein (Fig. 4F,G). In contrast, there was no change in expression of *Mmp9* mRNA (Fig. 4E) or protein following IR (Fig. 4F,H). The upregulation of MMP2 may also be linked to the edema-like morphological changes observed in the irradiated SMGs (Fig. S2C,D).

Similar to bilaterally irradiated SMGs, increased *Il1b* and *Tnf-α* mRNA levels were observed after unilateral IR using the SARRP (Fig. 4I,J). *Cxcl2* mRNA was also elevated at 3 h post-IR in comparison to controls (Fig. 4K). *Mmp2* mRNA expression was increased significantly at 24 and 48 h (Fig. 4L). Although both IR sources elicited similar pro-inflammatory responses, expression of these mRNAs was not significantly altered in the CL SMGs (Fig. 4I-L).

### IR transiently disrupts expression of mitochondrial factors

**Fig. 5.**
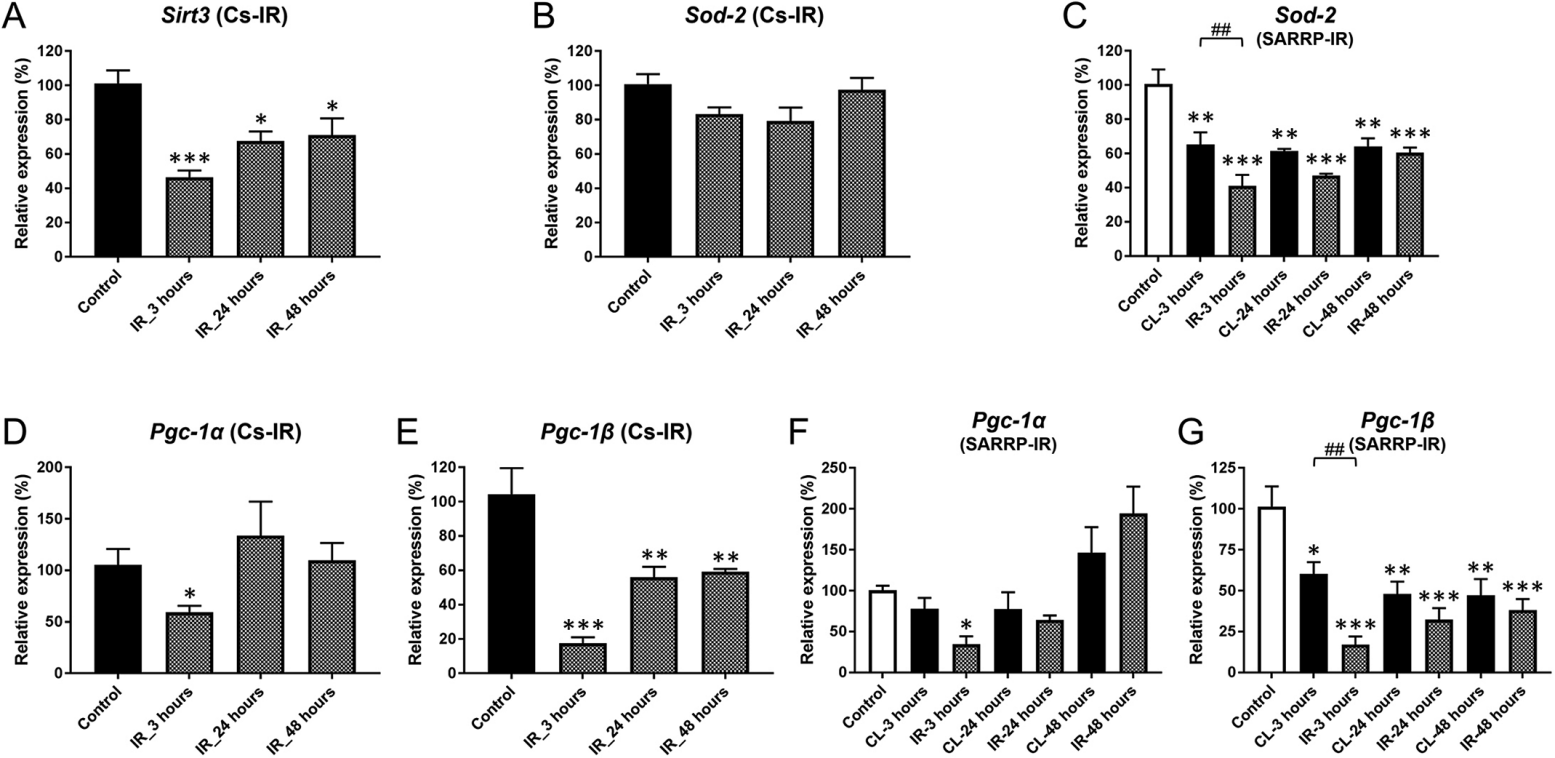
**IR transiently disrupts expression of mitochondrial factors.** (A) Expression of *Sirt3* was rapidly decreased by 3 h after IR with the Cs source (***P<0.001), compared to the non-irradiated control. *Sirt3* mRNA levels remained low at 24 and 48 h (*P<0.025). (B) *Sod2* mRNA levels did not significantly change after IR using the Cs source (one-way ANOVA; *F*=2.822, *P*=0.072). (C) In contrast, *Sod2* mRNA expression was significantly decreased after IR using SARRP, in both IR and CL SMGs by 3 h versus control (**P=0.001, ***P<0.001, respectively, versus control), with lower expression in the IR-treated SMG (^##^*P*<0.01). (D) Expression levels of *Pgc-1α* mRNA was decreased by 3 h (*P=0.037), but recovered by 24 h [*P*=0.997 (24 h), *P*=0.998 (48 h)]. (E) *Pgc-1β* mRNA expression was significantly decreased by 3 h (***P<0.001) in the irradiated SMGs and remained at a lower level than in the non-irradiated control up to 48 h (**P<0.004). (F) A similar expression profile for *Pgc-1α* mRNA (*P=0.027) was induced using SARRP. (G) IR using SARRP significantly reduced expression of *Pgc-1β* in both irradiated (***P<0.001 versus control) and CL (*P=0.012, **P=0.002) SMGs, with significantly lower expression in the IR-treated SMGs (^##^*P*<0.01 versus CL SMGs). Error bars indicate mean±s.e.m. Statistical analysis was performed compared to control (non-irradiated) using one-way ANOVA with Dunnett's post-hoc test, or comparing irradiated and CL SMGs using two-way ANOVA with Bonferroni test (*n*=3-5 each group).

Because IR rapidly disrupts mitochondrial function in SMGs (Liu et al., 2017; Kawamura et al., 2018), we used qPCR to look for changes in the expression of key factors involved in mitochondrial biogenesis or reactive oxygen species (ROS) regulation. *Sirt3*, which plays a central role in maintaining mitochondrial homeostasis after stress (Marcus and Andrabi, 2018), was transiently downregulated after IR, whereas *Sirt1* and *Sirt7*, which are localized to the nucleus and linked to DNA repair (Finkel et al., 2009; Vazquez et al., 2016), were not altered (Fig. 5A; Fig. S4E,F). mRNA expression of *Sod2*, encoding a mitochondrial superoxide dismutase that counters oxidative stress (Wang et al., 2018), did not change within 48 h after IR using the Cs source (Fig. 5B), but was significantly decreased in both SARRP-irradiated and CL SMGs (Fig. 5C). mRNA expression of *Sod1*, encoding a cytoplasmic protein, was not altered by IR (Fig. S4G,H). mRNA expression of the transcriptional co-activators PGC-1 alpha (*Pgc-1α*; also known as *Ppargc1a*) and PGC-1 beta (*Pgc-1β*; also known as *Ppargc1b*), key regulators of mitochondrial biogenesis, was downregulated within 3 h after IR administration from either source (Fig. 5D-G). In addition, *Pgc-1β* expression was significantly decreased in CL SMGs compared to control glands (Fig. 5G). Thus, IR both directly and indirectly disrupted expression of several mitochondrial factors.

## DISCUSSION

IR-induced effects in mouse salivary glands are divided into short- and long-term changes (Coppes et al., 2001; Jasmer et al., 2020). This study was undertaken to search for rapid changes in gene or protein expression that may yield insights into IR-induced causes of hyposalivation. Murine SMGs were irradiated bilaterally using a Cs irradiator, or unilaterally using the SARRP, an image-guided microirradiator. IR from the Cs source is in the form of gamma rays (with an energy of 662 keV), whereas the SARRP delivers 225 kVp X-rays with the beam filtered to remove low-energy photons. Both are categorized as low-linear energy transfer (LET) ionizing IR and result in similar, although not always identical, physiological effects (Iyer and Lehnert, 2000, 2002; International Agency for Research on Cancer, 2000). In this study, we observed that IR, whether applied unilaterally or bilaterally to the salivary glands, causes rapid and measurable responses that occur within 24-48 h. Our data show that changes in gene expression following bilateral IR of SMGs with the Cs source are similar to changes observed in SARRP-induced, unilaterally irradiated SMGs. Moreover, we observed that gene expression changes occurred in non-irradiated SMGs, indicating that they exhibit bystander effects.

We generated a profile of the IR-induced loss of saliva secretion over time following IR treatment. In agreement with earlier studies (Avila et al., 2009; Grundmann et al., 2010; Morgan-Bathke et al., 2014), we measured a significant decrease in saliva volume at 3 h following bilateral IR and observed that secretion recovered by 24 h. At 48 h, secretion was again decreased and, after a modest recovery at 1-2 weeks, saliva volumes continued to decline.

Increased extracellular space and fluid accumulation seen in SMGs after IR suggest disruption of the epithelial barrier. Alterations in expression of the tight junction genes claudin-3 and claudin-4 occurs within 3 days of IR in parotid glands (Yokoyama et al., 2017), and increased claudin-4 expression accompanies acute lung injury (Wray et al., 2009). We found that claudin-3 and claudin-4 mRNAs were upregulated as early as 3 h post-IR, which should preserve intracellular junctions. However, claudins promote the activation of pro-MMP2 (Miyamori et al., 2001; Lee et al., 2008; Hwang et al., 2010), which degrades type IV collagen, a major component of the basement membrane (Araya et al., 2001; Zhao et al., 2004). MMP2 was increased in minipig parotid glands (Lombaert et al., 2020) and in IR-treated SMGs at 24 h, consistent with increased intercellular edema.

Consistent with previous studies (Choi et al., 2009; Marmary et al., 2016), we did not detect an increase in apoptosis following IR, although the number of γH2AX foci increased significantly. IR induces the activation of p53 and the pro-apoptotic factors BAX and PUMA by 4 h post-IR in salivary glands (Limesand et al., 2006; Avila et al., 2009), but levels of apoptosis remain low and decline further by 48 h (Avila et al., 2009). BAX homodimers accelerate apoptosis by inducing release of cytochrome c, whereas heterodimers of BAX with the anti-apoptotic proteins Bcl-2 or Bcl-xl prevent caspase activation (Reed, 1994; Kim, 2005). We found that Bcl-2 and Bcl-xl were both upregulated within 3 h post-IR, as was p21. The transient upregulation of these anti-apoptotic factors, together with p21, which functions to suppress apoptosis in part by inhibiting the activity of caspases (Sohn et al., 2006; Mirzayans et al., 2013), may limit apoptosis. Thus, in contrast to other IR-sensitive tissues (Ding et al., 2016), IR of the SMGs appears to favor the damage repair response, rather than apoptosis.

IR induced a transient decrease in the transcription factor MIST1, which maintains the secretory phenotype of acinar cells (Pin et al., 2001; Lo et al., 2017). MIST1 regulates the expression of *Aqp5*, which encodes a water channel critical for saliva secretion (Jia et al., 2008), and *Cx32*, which encodes a gap junction protein in exocrine acinar cells (Rukstalis et al., 2003). The sensitivity of the MIST1 transcription factor to stress or injury (Karki et al., 2015) most likely accounts for the downregulation of all three mRNAs at 3 h post-IR. Consistent with earlier reports in rat SMGs (Takagi et al., 2003; Li et al., 2006), AQP5 protein levels decreased within 48 h.

IR has been linked to disruptions in calcium signaling and mitochondrial pathways in acinar cells (Liu et al., 2017). Sirtuins are histone deacetylases, which influence cellular responses to external signals by regulating cell cycle, metabolism and genome stability (Chalkiadaki and Guarente, 2015). A previous study found that *Sirt1* mRNA was significantly increased in mouse parotid glands within 30 min after IR with 5 Gy (Meyer et al., 2017). However, there was no significant change in *Sirt1* or *Sirt7* mRNA levels in SMGs at 3 h after 15 Gy IR.​ The discrepancy may be due to different IR dose, time of analysis or salivary gland type. In contrast, *Sirt3*, encoding a mitochondrial protein that coordinates mitochondrial metabolism (Giralt and Villarroya, 2012), thereby limiting levels of ROS (Chalkiadaki and Guarente, 2015), was transiently downregulated at 3 h post-IR. The expression of SOD genes involved in ROS homeostasis (Zelko et al., 2002) was not changed, but the co-activators PGC-1α and PGC-1β, which regulate the SOD genes (Lin et al., 2005), were downregulated by 3 h post-IR. Further investigation into gene expression changes within these rapidly responsive pathways is warranted.

ROS produced by low-LET IR can migrate to distant sites through cell contacts, or across cell membranes, causing responses in non-targeted cells (Desouky et al., 2015). The precision of SARRP allowed us to investigate off-target effects induced in the CL SMGs. Unilateral IR resulted in gene expression changes not only in the irradiated, but also in the CL SMGs. Similar off-target effects were observed in the non-irradiated parotid glands of minipigs (Lombaert et al., 2020). In addition to ROS, release of soluble factors, including nitric oxide and activated cytokines, from irradiated cells contributes to bystander effects (Prise and O'Sullivan, 2009; Najafi et al., 2014). We speculate that the elevated expression of the cytokines IL-1β, TNF-α and Cxcl2 by 3 h after IR is involved in the propagation of bystander effects to the CL SMGs. Although not investigated in this study, purinergic receptors are also likely to play a role in promoting bystander effects (Jasmer et al., 2020).

Importantly, our results demonstrate that non-irradiated CL SMGs undergo many of the changes in gene expression that are detected in the unilaterally irradiated SMGs. Although the coupling mechanism is not understood, it has been reported that unilateral injury or stress results in a similar response in both SMGs (Walker and Gobé, 1987; Lombaert et al., 2020). Thus, investigations into how IR impacts salivary gland function must take into account the bystander effects on non-irradiated glands. The rational design of radioprotective strategies will require elucidation of the mechanisms involved.

## MATERIALS AND METHODS

### Animals

Female C57BL/6J (The Jackson Laboratory, Bar Harbor, ME, USA) mice aged 4-12 weeks old were used in this study. Animals were housed in groups and maintained on a 12 h light/dark cycle with food and water available *ad libitum*. All procedures were approved and conducted in accordance with the University Committee on Animal Resources at the University of Rochester Medical Center.

### IR

Two IR sources were utilized to investigate the short-term effects of IR. The Cs IR source (Shepherd Mark I ^137^Cs gamma ray irradiator), in combination with a custom-built brain-slit collimator of 4 mm, was used to deliver IR bilaterally, but to limit IR exposure to the neck region of C57BL/6 female mice (Fig. S1A), as previously described (Weng et al., 2018). Mice were anesthetized with ketamine (90 mg/kg) and xylazine (9 mg/kg) via intraperitoneal injection and irradiated with a single dose of 15.0 Gy within 20 min. Control mice were administered ketamine/xylazine, but not irradiated.

The SARRP (Xstrahl, Suwanee, GA, USA) permits unilateral IR of a single SMG (Fig. S1B,C), through spatial targeting of the IR area following a CT scan (Fig. S1D-F). Mice were anesthetized with vaporized isoflurane through a nose cone, and a single dose of 15.0 Gy was targeted to the left SMG of irradiated mice, using a 10×10 mm collimator, as previously described (Bachman et al., 2020).

Mice irradiated with the Cs source were analyzed for saliva secretion, histology and gene expression shortly after IR. Mice irradiated using the SARRP were analyzed for histology and gene expression post-IR.

### Saliva collection

Total saliva was collected both before and after IR from the same mice. To establish the baseline of saliva volume, mice were anesthetized with ketamine (75 mg/kg) and xylazine (7.5 mg/kg), and their saliva secretion was stimulated with an intraperitoneal injection of the muscarinic receptor antagonist pilocarpine (0.5 mg/kg). Total whole saliva was collected for 20 min into pre-weighed tubes using glass capillary tubes placed into the oral cavity under the tongue. Total saliva volume was normalized to individual body weight (μl/g) at each time point.

### Histological analysis

SMGs were dissected from control (non-irradiated), Cs-irradiated and SARRP-irradiated (both irradiated and CL SMGs) mice at 3, 24 and 48 h post-IR and fixed in 4% paraformaldehyde at 4°C overnight. Mice used for saliva collection were not used for tissue collection. Staining was processed as previously described (Varghese et al., 2018; Weng et al., 2018). Briefly, fixed tissues were embedded in paraffin, and sections were cut to 5 μm and stained with Hematoxylin and Eosin (H&E) for morphological assessment. For immunohistochemistry, antigen retrieval was performed in HIER buffer (10 mM Tris-base, 1 mM EDTA, pH 9.4) or citrate buffer, pH 6.0. CAS-Block^TM^ Histochemical Reagent (008120, Thermo Fisher Scientific) was used to block for 1 h. Primary antibodies anti-AQP5 (ab92320, Abcam, 1:100), anti-CLN3 (ab15102, Abcam, 1:500), anti-CLN4 (ab53156, Abcam, 1:500), anti-γH2AX (05-636, Millipore Sigma, 1:200), anti-MIST1 (ab187978, Abcam, 1:200), anti-M3R (M0194, Sigma-Aldrich, 1:200), anti-NKCC1 (85403, Cell Signaling, 1:1000), anti-ZO-1 (33-9100, Thermo Fisher Scientific, 1:1000) were applied overnight at 4°C. Secondary antibodies were donkey anti-rabbit immunoglobulin G (IgG) Alexa Fluor 488 and 594 (A21206 and A21207, respectively, Thermo Fisher Scientific, 1:1000) or donkey anti-mouse IgG Alexa Fluor 488 and 594 (A21202 and A21203, respectively, Thermo Fisher Scientific, 1:1000). Nuclei were stained with 4′,6-diamidino-2-phenylindole (DAPI; D1306, Thermo Fisher Scientific).

Imaging and analysis were performed as previously described (Weng et al., 2018; Ingalls et al., 2020). Images for H&E staining were acquired using an Olympus DX41 microscope with a DP41 camera and analyzed using ImageJ software [National Institutes of Health (NIH)]. Fluorescent images were acquired using an Olympus IX85 phase-contrast microscope at 40× magnification or a Leica TCS SP5 confocal microscope with 40× oil immersion objective and Argon laser. Fluorescent images were converted to 8-bit format, thresholded to binary, and a watershed function was used to distinguish individual cells. The number of nuclei (DAPI^+^, NKCC1^+^ and NKCC1^−^) and dots (γH2AX^+^) were counted automatically using the particle analysis plugin.

### Total RNA extraction and qPCR

SMGs were dissected from control (non-irradiated), Cs-irradiated and SARRP-irradiated (both IR and CL SMGs) mice at 3, 24 and 48 h post-IR. SMGs were dissected into TRIzol reagent and stored at −80°C until total RNA extraction. Total RNA was extracted using an E.Z.N.A. Total RNA kit (R6834, Omega Bio-tek) and reverse transcribed using an iScript^TM^ cDNA synthesis kit (Bio-Rad), according to the manufacturers' instructions. qPCR analysis of individual cDNAs was processed on a CFX96TM Real-Time System (Bio-Rad) using SsoAdvanced^TM^ Universal SYBR Green Supermix (Bio-Rad) and the following PCR primer sets: mouse *Rps29* (reference gene), *Mist1*, *Aqp5*, *M3r*, *P2y2*, *Il1b*, *Tnf-α*, *Cxcl2*, *Mmp2*, *Bax*, *Bcl2*, *Bcl-xl*, *p21*, *Sirt1*, *Sirt3*, *Sirt7*, *Foxo3a*, *Gadd45a*, *Tgfb1*, *Sod1*, *Sod2*, *Pgc-1α* and *Pgc-1β* (primer sequences are listed in Table S1). Target genes were normalized to mouse *Rps29* as a reference gene. Reference and target genes were only compared from the same plate. qPCR results were analyzed by the 2^–▵▵Ct^ method. C_t_ values of less than 35 were obtained from all target genes. All genes were measured from *n*=3-5 mice. cDNA samples were tested using biological duplicates (two wells per primer set per sample). A series experiment [control, 3, 24 and 48 h post-IR using Cs source, or control (non-irradiated), CL and irradiated SMGs at 3, 24 and 48 h post-IR using SARRP] was run using several primer sets.

### Western blot analysis

Total protein was extracted from the SMGs of control (non-irradiated) and Cs-irradiated mice at 3, 24 and 24 h post-IR as previously described (Li et al., 2006). Briefly, isolated SMGs were placed in chilled protein extraction buffer [50 mM Tris-HCl (pH 8.0), 2 mM EDTA, 250 mM sucrose, 1 mM β-mercaptoethanol and protease/phosphatase inhibitor; Cell Signaling Technology]. The tissue was homogenized for 1 min on ice. The homogenate was centrifuged at 369 ***g*** for 15 min at 4°C, and the supernatant was collected as the total protein fraction. The protein concentration of each sample was measured at 750 nm by using Protein Assay Dye Reagent (5000006, BioRad) according to the manufacturer's protocol. Then, 20 µg of total protein was run on a MINI-PROTEIN TGX pre-cast gel (4561093, Bio-Rad), followed by protein transfer onto PVDF membrane (1620174, Bio-Rad). The membrane was blocked in 2% bovine serum albumin (BSA)-1× Tris-buffered saline, 0.1% Tween 20 (TBST) or 5% skim milk-TBST at room temperature for 1 h. The following primary antibodies were applied overnight at 4°C: anti-AQP5 (ab92320, Abcam, 1:1000), anti-CLN3 (34-1700, Thermo Fisher Scientific, 1:1000), anti-CLN4 (ab53156, Abcam, 1:1000), anti-CX32 (13-8200, Thermo Fisher Scientific, 1:1000), anti-E-CAD (610181, BD Biosciences, 1:3000), anti-MIST1 (ab187978, Abcam, 1:1000), anti-MMP2 (10373-2-AP, Thermo Fisher Scientific, 1:1000), anti-MMP9 (10375-2-AP, Thermo Fisher Scientific, 1:1000), anti-β-actin (sc-47778, Santa Cruz Biotechnology, 1:3000) and anti-GAPDH (AM4300, Thermo Fisher Scientific, 3 µg/ml). Appropriate secondary antibodies [anti-rabbit IgG (MP-7401, Vector Laboratories) or anti-mouse IgG (1706516, Bio-Rad)] were hybridized to detect the proteins. The signals were detected using Pierce^TM^ ECL Western Blotting Substrate (32106, Thermo Fisher Scientific). For the re-probing of loading control (β-actin or GAPDH), the stained membrane was washed with TBST and incubated in Restore Western Blot Stripping Buffer (21059, Thermo Fisher Scientific) for 30 min at room temperature. After three washes with TBST, the membrane was blocked in 2% BSA-TBST or 5% skim milk-TBST at room temperature for 1 h. The primary antibody (anti-β-actin or anti-GAPDH) was applied overnight at 4°C. The membrane was incubated with anti-mouse IgG (1706516, Bio-Rad) secondary antibody, and protein was detected using Pierce^TM^ ECL Western Blotting Substrate (32106, Thermo Fisher Scientific). Protein levels were quantified and compared using ImageJ (NIH) densitometric analysis (Schneider et al., 2012).

### Statistical analysis

All results are presented as mean±s.d. Data for bilateral IR were analyzed by one-way analysis of variance (ANOVA) with Dunnett's post-hoc test compared to control (non-irradiated) SMGs. Two-way ANOVA with Bonferroni post-hoc test was used to compare the CL and SARRP-irradiated SMGs using SPSS. Differences were considered significant at *P*<0.05.

## Supplementary Material

10.1242/dmm.049570_sup1 Supplementary information

## References

[DMM049570C1] Araya, J., Maruyama, M., Sassa, K., Fujita, T., Hayashi, R., Matsui, S., Kashii, T., Yamashita, N., Sugiyama, E. and Kobayashi, M. (2001). Ionizing radiation enhances matrix metalloproteinase-2 production in human lung epithelial cells. *Am. J. Physiol. Lung Cell. Mol. Physiol.* 280, L30-L38. 10.1152/ajplung.2001.280.1.L30 11133492

[DMM049570C2] Avila, J. L., Grundmann, O., Burd, R. and Limesand, K. H. (2009). Radiation-induced salivary gland dysfunction results from p53-dependent apoptosis. *Int. J. Radiat. Oncol. Biol. Phys.* 73, 523-529. 10.1016/j.ijrobp.2008.09.036 19147016 PMC2631421

[DMM049570C3] Bachman, J. F., Blanc, R. S., Paris, N. D., Kallenbach, J. G., Johnston, C. J., Hernady, E., Williams, J. P. and Chakkalakal, J. V. (2020). Radiation-induced damage to prepubertal Pax7^+^ skeletal muscle stem cells drives lifelong deficits in myofiber size and nuclear number. *iScience* 23, 101760. 10.1016/j.isci.2020.101760 33241204 PMC7674517

[DMM049570C4] Baker, O. J. (2016). Current trends in salivary gland tight junctions. *Tissue Barriers* 4, e1162348. 10.1080/21688370.2016.1162348 27583188 PMC4993573

[DMM049570C6] Blyth, B. J. and Sykes, P. J. (2011). Radiation-induced bystander effects: what are they, and how relevant are they to human radiation exposures? *Radiat. Res.* 176, 139-157. 10.1667/RR2548.1 21631286

[DMM049570C8] Chalkiadaki, A. and Guarente, L. (2015). The multifaceted functions of sirtuins in cancer. *Nat. Rev. Cancer* 15, 608-624. 10.1038/nrc3985 26383140

[DMM049570C9] Choi, J. H., Wu, H.-G., Jung, K. C., Lee, S. H. and Kwon, E. K. (2009). Apoptosis and expression of AQP5 and TGF-beta in the irradiated rat submandibular gland. *Cancer Res. Treat* 41, 145-154. 10.4143/crt.2009.41.3.145 19809564 PMC2757666

[DMM049570C10] Coppes, R. P., Zeilstra, L. J., Kampinga, H. H. and Konings, A. W. (2001). Early to late sparing of radiation damage to the parotid gland by adrenergic and muscarinic receptor agonists. *Br. J. Cancer* 85, 1055-1063. 10.1054/bjoc.2001.2038 11592779 PMC2375094

[DMM049570C11] Cramer, J. D., Burtness, B., Le, Q. T. and Ferris, R. L. (2019). The changing therapeutic landscape of head and neck cancer. *Nat. Rev. Clin. Oncol.* 16, 669-683. 10.1038/s41571-019-0227-z 31189965

[DMM049570C13] Daguenet, E., Louati, S., Wozny, A. S., Vial, N., Gras, M., Guy, J. B., Vallard, A., Rodriguez-Lafrasse, C. and Magné, N. (2020). Radiation-induced bystander and abscopal effects: important lessons from preclinical models. *Br. J. Cancer* 123, 339-348. 10.1038/s41416-020-0942-3 32581341 PMC7403362

[DMM049570C14] Desouky, O., Ding, N. and Zhou, G. (2015). Targeted and non-targeted effects of ionizing radiation. *J. Radiat. Res. Appl. Sci.* 8, 247-254. 10.1016/j.jrras.2015.03.003

[DMM049570C15] Ding, D., Zhang, Y., Wang, J., Zhang, X., Gao, Y., Yin, L., Li, Q., Li, J. and Chen, H. (2016). Induction and inhibition of the pan-nuclear gamma-H2AX response in restiing human peripheral blood lymphocytes after X-ray irradiation. *Cell Death Discov.* 2, 16011. 10.1038/cddiscovery.2016.11 27551505 PMC4979483

[DMM049570C16] Duarte, S., Baber, J., Fujii, T. and Coito, A. J. (2015). Matrix metalloproteinases in liver injury, repair and fibrosis. *Matrix Biol.* 44-46, 147-156. 10.1016/j.matbio.2015.01.004 25599939 PMC4495728

[DMM049570C17] Finkel, T., Deng, C. X. and Mostoslavsky, R. (2009). Recent progress in the biology and physiology of sirtuins. *Nature* 460, 587-591. 10.1038/nature08197 19641587 PMC3727385

[DMM049570C18] Giralt, A. and Villarroya, F. (2012). SIRT3, a pivotal actor in mitochondrial functions: metabolism, cell death and aging. *Biochem. J.* 444, 1-10. 10.1042/BJ20120030 22533670

[DMM049570C19] Grundmann, O., Fillinger, J. L., Victory, K. R., Burd, R. and Limesand, K. H. (2010). Restoration of radiation therapy-induced salivary gland dysfunction in mice by post therapy IGF-1 administration. *BMC Cancer* 10, 417. 10.1186/1471-2407-10-417 20698985 PMC3087323

[DMM049570C21] Hwang, T. L., Lee, L. Y., Wang, C. C., Liang, Y., Huang, S. F. and Wu, C. M. (2010). Claudin-4 expression is associated with tumor invasion, MMP-2 and MMP-9 expression in gastric cancer. *Exp. Ther. Med.* 1, 789-797. 10.3892/etm.2010.116 22993603 PMC3445934

[DMM049570C22] Ingalls, M. H., Hollomon, A. J., Newlands, S. D., McDavid, A. N. and Ovitt, C. E. (2020). Intrinsic mitotic activity supports the human salivary gland acinar cell population. *FEBS Lett.* 594, 376-382. 10.1002/1873-3468.13611 31538335 PMC6987008

[DMM049570C23] International Agency for Research on Cancer (2000). *Ionizing Radiation, Part 1: X- and Gamma-Radiation, and Neutrons. Overall Introduction. IARC Monogr Eval Carcinog Risks Hum*, Vol. 75 Pt 1, pp. 35-37. IARC Publications. 10932818 PMC5220263

[DMM049570C24] Iyer, R. and Lehnert, B. E. (2000). Effects of ionizing radiation in targeted and nontargeted cells. *Arch. Biochem. Biophys.* 376, 14-25. 10.1006/abbi.1999.1684 10729186

[DMM049570C25] Iyer, R. and Lehnert, B. E. (2002). Low dose, low-LET ionizing radiation-induced radioadaptation and associated early responses in unirradiated cells. *Mutat. Res.* 503, 1-9. 10.1016/S0027-5107(02)00068-4 12052498

[DMM049570C26] Jasmer, K. J., Gilman, K. E., Muñoz Forti, K., Weisman, G. A. and Limesand, K. H. (2020). Radiation-induced salivary gland dysfunction: mechanisms, therapeutics and future directions. *J. Clin. Med.* 9, 4095. 10.3390/jcm9124095 33353023 PMC7767137

[DMM049570C27] Jia, D., Sun, Y. and Konieczny, S. F. (2008). Mist1 regulates pancreatic acinar cell proliferation through p21 CIP1/WAF1. *Gastroenterology* 135, 1687-1697. 10.1053/j.gastro.2008.07.026 18762186 PMC2853247

[DMM049570C28] Karki, A., Humphrey, S. E., Steele, R. E., Hess, D. A., Taparowsky, E. J. and Konieczny, S. F. (2015). Silencing Mist1 gene expression is essential for recovery from acute pancreatitis. *PLoS One* 10, e0145724. 10.1371/journal.pone.0145724 26717480 PMC4696804

[DMM049570C30] Kawamura, K., Qi, F. and Kobayashi, J. (2018). Potential relationship between the biological effects of low-dose irradiation and mitochondrial ROS production. *J. Radiat. Res.* 59, ii91-ii97. 10.1093/jrr/rrx091 29415254 PMC5941154

[DMM049570C31] Khalafalla, M. G., Woods, L. T., Jasmer, K. J., Forti, K. M., Camden, J. M., Jensen, J. L., Limesand, K. H., Galtung, H. K. and Weisman, G. A. (2020). P2 Receptors as therapeutic targets in the salivary gland: from physiology to dysfunction. *Front. Pharmacol.* 11, 222. 10.3389/fphar.2020.00222 32231563 PMC7082426

[DMM049570C32] Kim, R. (2005). Unknotting the roles of Bcl-2 and Bcl-xL in cell death. *Biochem. Biophys. Res. Commun.* 333, 336-343. 10.1016/j.bbrc.2005.04.161 15922292

[DMM049570C33] Konings, A. W., Coppes, R. P. and Vissink, A. (2005). On the mechanism of salivary gland radiosensitivity. *Int. J. Radiat. Oncol. Biol. Phys.* 62, 1187-1194. 10.1016/j.ijrobp.2004.12.051 15990024

[DMM049570C34] Lee, L. Y., Wu, C. M., Wang, C. C., Yu, J. S., Liang, Y., Huang, K. H., Lo, C. H. and Hwang, T. L. (2008). Expression of matrix metalloproteinases MMP-2 and MMP-9 in gastric cancer and their relation to claudin-4 expression. *Histol. Histopathol.* 23, 515-521. 10.14670/HH-23.515 18283635

[DMM049570C35] Li, Z., Zhao, D., Gong, B., Xu, Y., Sun, H., Yang, B. and Zhao, X. (2006). Decreased saliva secretion and down-regulation of AQP5 in submandibular gland in irradiated rats. *Radiat. Res.* 165, 678-687. 10.1667/RR3569.1 16802868

[DMM049570C37] Limesand, K. H., Schwertfeger, K. L. and Anderson, S. M. (2006). MDM2 is required for suppression of apoptosis by activated Akt1 in salivary acinar cells. *Mol. Cell. Biol.* 26, 8840-8856. 10.1128/MCB.01846-05 16982679 PMC1636839

[DMM049570C38] Lin, J., Handschin, C. and Spiegelman, B. M. (2005). Metabolic control through the PGC-1 family of transcription coactivators. *Cell Metab.* 1, 361-370. 10.1016/j.cmet.2005.05.004 16054085

[DMM049570C39] Liu, X., Gong, B., de Souza, L. B., Ong, H. L., Subedi, K. P., Cheng, K. T., Swaim, W., Zheng, C., Mori, Y. and Ambudkar, I. S. (2017). Radiation inhibits salivary gland function by promoting STIM1 cleavage by caspase-3 and loss of SOCE through a TRPM2-dependent pathway. *Sci. Signal.* 10, eaal4064. 10.1126/scisignal.aal4064 28588080 PMC5798857

[DMM049570C40] Lo, H. G., Jin, R. U., Sibbel, G., Liu, D., Karki, A., Joens, M. S., Madison, B. B., Zhang, B., Blanc, V., Fitzpatrick, J. A. et al. (2017). A single transcription factor is sufficient to induce and maintain secretory cell architecture. *Genes Dev.* 31, 154-171. 10.1101/gad.285684.116 28174210 PMC5322730

[DMM049570C41] Lombaert, I. M. A., Patel, V. N., Jones, C. E., Villier, D. C., Canada, A. E., Moore, M. R., Berenstein, E., Zheng, C., Goldsmith, C. M., Chorini, J. A. et al. (2020). CERE-120 prevents irradiation-induced hypofunction and restores immune homeostasis in porcine salivary glands. *Mol. Ther. Methods Clin. Dev.* 18, 839-855. 10.1016/j.omtm.2020.07.016 32953934 PMC7479444

[DMM049570C42] Löbrich, M., Shibata, A., Beucher, A., Fisher, A., Ensminger, M., Goodarzi, A. A., Barton, O. and Jeggo, P. A. (2010). gammaH2AX foci analysis for monitoring DNA double-strand break repair: strengths, limitations and optimization. *Cell Cycle* 9, 662-669. 10.4161/cc.9.4.10764 20139725

[DMM049570C44] Marcus, J. M. and Andrabi, S. A. (2018). SIRT3 regulation under cellular stress: making sense of the ups and downs. *Front. Neurosci.* 12, 799. 10.3389/fnins.2018.00799 30450031 PMC6224517

[DMM049570C45] Marmary, Y., Adar, R., Gaska, S., Wygoda, A., Maly, A., Cohen, J., Eliashar, R., Mizrachi, L., Orfaig-Geva, C., Baum, B. J. et al. (2016). Radiation-induced loss of salivary gland function is driven by cellular senescence and prevented by IL6 modulation. *Cancer Res.* 76, 1170-1180. 10.1158/0008-5472.CAN-15-1671 26759233

[DMM049570C46] Meyer, S., Chibly, A. M., Burd, R. and Limesand, K. H. (2017). Insulin-like growth factor-1-mediated DNA repair in irradiated salivary glands is Sirtuin-1 dependent. *J. Dent. Res.* 96, 225-232. 10.1177/0022034516677529 28106504 PMC5331616

[DMM049570C47] Mirzayans, R., Andrais, B., Scott, A., Wang, Y. W. and Murray, D. (2013). Ionizing radiation-induced responses in human cells with differing TP53 status. *Int. J. Mol. Sci.* 14, 22409-22435. 10.3390/ijms141122409 24232458 PMC3856071

[DMM049570C48] Miyamori, H., Takino, T., Kobayashi, Y., Tokai, H., Itoh, Y., Seiki, M. and Sato, H. (2001). Claudin promotes activation of pro-matrix metalloproteinase-2 mediated by membrane-type matrix metalloproteinases. *J. Biol. Chem.* 276, 28204-28211. 10.1074/jbc.M103083200 11382769

[DMM049570C50] Morgan-Bathke, M., Hill, G. A., Harris, Z. I., Lin, H. H., Chibly, A. M., Klein, R. R., Burd, R., Ann, D. K. and Limesand, K. H. (2014). Autophagy correlates with maintenance of salivary gland function following radiation. *Sci. Rep.* 4, 5206. 10.1038/srep05206 24903000 PMC4047540

[DMM049570C51] Najafi, M., Fardid, R., Hadadi, G. and Fardid, M. (2014). The mechanisms of radiation-induced bystander effect. *J. Biomed. Phys. Eng.* 4, 163-172. 25599062 PMC4289523

[DMM049570C52] Pin, C. L., Bonvissuto, A. C. and Konieczny, S. F. (2000). Mist1 expression is a common link among serous exocrine cells exhibiting regulated exocytosis. *Anat. Rec.* 259, 157-167. 10.1002/(SICI)1097-0185(20000601)259:2<157::AID-AR6>3.0.CO;2-0 10820318

[DMM049570C53] Pin, C. L., Rukstalis, J. M., Johnson, C. and Konieczny, S. F. (2001). The bHLH transcription factor Mist1 is required to maintain exocrine pancreas cell organization and acinar cell identity. *J. Cell Biol.* 155, 519-530. 10.1083/jcb.200105060 11696558 PMC2198859

[DMM049570C54] Pinna, R., Campus, G., Cumbo, E., Mura, I. and Milia, E. (2015). Xerostomia induced by radiotherapy: an overview of the physiopathology, clinical evidence, and management of the oral damage. *Ther. Clin. Risk Manag.* 11, 171-188. 10.2147/TCRM.S70652 25691810 PMC4325830

[DMM049570C55] Prise, K. M. and O'Sullivan, J. M. (2009). Radiation-induced bystander signalling in cancer therapy. *Nat. Rev. Cancer* 9, 351-360. 10.1038/nrc2603 19377507 PMC2855954

[DMM049570C56] Reed, J. C. (1994). Bcl-2 and the regulation of programmed cell death. *J. Cell Biol.* 124, 1-6. 10.1083/jcb.124.1.1 8294493 PMC2119888

[DMM049570C57] Riley, T., Sontag, E., Chen, P. and Levine, A. (2008). Transcriptional control of human p53-regulated genes. *Nat. Rev. Mol. Cell Biol.* 9, 402-412. 10.1038/nrm2395 18431400

[DMM049570C60] Rukstalis, J. M., Kowalik, A., Zhu, L., Lidington, D., Pin, C. L. and Konieczny, S. F. (2003). Exocrine specific expression of Connexin32 is dependent on the basic helix-loop-helix transcription factor Mist1. *J. Cell Sci.* 116, 3315-3325. 10.1242/jcs.00631 12829745

[DMM049570C62] Schneider, C. A., Rasband, W. S. and Eliceiri, K. W. (2012). NIH Image to ImageJ: 25 years of image analysis. *Nat. Methods* 9, 671-675. 10.1038/nmeth.2089 22930834 PMC5554542

[DMM049570C63] Shen, Z., Wang, J., Huang, Q., Shi, Y., Wei, Z., Zhang, X., Qiu, Y., Zhang, M., Wang, Y., Qin, W. et al. (2018). Genetic modification to induce CXCR2 overexpression in mesenchymal stem cells enhances treatment benefits in radiation-induced oral mucositis. *Cell Death Dis.* 9, 229. 10.1038/s41419-018-0310-x 29445104 PMC5833705

[DMM049570C66] Sohn, D., Essmann, F., Schulze-Osthoff, K. and Jänicke, R. U. (2006). p21 blocks irradiation-induced apoptosis downstream of mitochondria by inhibition of cyclin-dependent kinase-mediated caspase-9 activation. *Cancer Res.* 66, 11254-11262. 10.1158/0008-5472.CAN-06-1569 17145870

[DMM049570C69] Takagi, K., Yamaguchi, K., Sakurai, T., Asari, T., Hashimoto, K. and Terakawa, S. (2003). Secretion of saliva in X-irradiated rat submandibular glands. *Radiat. Res.* 159, 351-360. 10.1667/0033-7587(2003)159[0351:SOSIXI]2.0.CO;2 12600238

[DMM049570C70] Turner, J. T., Weisman, G. A. and Camden, J. M. (1997). Upregulation of P2Y2 nucleotide receptors in rat salivary gland cells during short-term culture. *Am. J. Physiol.* 273, C1100-C1107. 10.1152/ajpcell.1997.273.3.C1100 9316432

[DMM049570C71] Varghese, J. J., Schmale, I. L., Mickelsen, D., Hansen, M. E., Newlands, S. D., Benoit, D. S. W., Korshunov, V. A. and Ovitt, C. E. (2018). Localized delivery of amifostine enhances salivary gland radioprotection. *J. Dent. Res.* 97, 1252-1259. 10.1177/0022034518767408 29634396 PMC6151913

[DMM049570C72] Vazquez, B. N., Thackray, J. K., Simonet, N. G., Kane-Goldsmith, N., Martinez-Redondo, P., Nguyen, T., Bunting, S., Vaquero, A., Tischfield, J. A. and Serrano, L. (2016). SIRT7 promotes genome integrity and modulates non-homologous end joining DNA repair. *EMBO J.* 35, 1488-1503. 10.15252/embj.201593499 27225932 PMC4884211

[DMM049570C73] Villa, A., Connell, C. L. and Abati, S. (2015). Diagnosis and management of xerostomia and hyposalivation. *Ther. Clin. Risk Manag.* 11, 45-51. 10.2147/TCRM.S76282 25653532 PMC4278738

[DMM049570C74] Vissink, A., Mitchell, J. B., Baum, B. J., Limesand, K. H., Jensen, S. B., Fox, P. C., Elting, L. S., Langendijk, J. A., Coppes, R. P. and Reyland, M. E. (2010). Clinical management of salivary gland hypofunction and xerostomia in head-and-neck cancer patients: successes and barriers. *Int. J. Radiat. Oncol. Biol. Phys.* 78, 983-991. 10.1016/j.ijrobp.2010.06.052 20970030 PMC2964345

[DMM049570C75] Vissink, A., van Luijk, P., Langendijk, J. A. and Coppes, R. P. (2015). Current ideas to reduce or salvage radiation damage to salivary glands. *Oral Dis.* 21, e1-e10. 10.1111/odi.12222 24581290

[DMM049570C76] Walker, N. I. and Gobé, G. C. (1987). Cell death and cell proliferation during atrophy of the rat parotid gland induced by duct obstruction. *J. Pathol.* 153, 333-344. 10.1002/path.1711530407 3430235

[DMM049570C77] Wang, J. L., Sun, Y. and Wu, S. (2000). Gamma-irradiation induces matrix metalloproteinase II expression in a p53-dependent manner. *Mol. Carcinog.* 27, 252-258. 10.1002/(SICI)1098-2744(200004)27:4<252::AID-MC2>3.0.CO;2-3 10747288

[DMM049570C78] Wang, Y., Branicky, R., Noë, A. and Hekimi, S. (2018). Superoxide dismutases: dual roles in controlling ROS damage and regulating ROS signaling. *J. Cell Biol.* 217, 1915-1928. 10.1083/jcb.201708007 29669742 PMC5987716

[DMM049570C79] Weng, P. L., Aure, M. H., Maruyama, T. and Ovitt, C. E. (2018). Limited regeneration of adult salivary glands after severe injury involves cellular plasticity. *Cell Rep.* 24, 1464-1470.e3. 10.1016/j.celrep.2018.07.016 30089258 PMC6350767

[DMM049570C80] Wong, W. Y., Pier, M. and Limesand, K. H. (2018). Persistent disruption of lateral junctional complexes and actin cytoskeleton in parotid salivary glands following radiation treatment. *Am. J. Physiol. Regul. Integr. Comp. Physiol.* 315, R656-R667. 10.1152/ajpregu.00388.2017 29897817 PMC6230885

[DMM049570C81] Wray, C., Mao, Y., Pan, J., Chandrasena, A., Piasta, F. and Frank, J. A. (2009). Claudin-4 augments alveolar epithelial barrier function and is induced in acute lung injury. *Am. J. Physiol. Lung Cell. Mol. Physiol.* 297, L219-L227. 10.1152/ajplung.00043.2009 19447895 PMC2742793

[DMM049570C83] Yokoyama, M., Narita, T., Sakurai, H., Katsumata-Kato, O., Sugiya, H. and Fujita-Yoshigaki, J. (2017). Maintenance of claudin-3 expression and the barrier functions of intercellular junctions in parotid acinar cells via the inhibition of Src signaling. *Arch. Oral Biol.* 81, 141-150. 10.1016/j.archoralbio.2017.05.007 28528309

[DMM049570C84] Zelko, I. N., Mariani, T. J. and Folz, R. J. (2002). Superoxide dismutase multigene family: a comparison of the CuZn-SOD (SOD1), Mn-SOD (SOD2), and EC-SOD (SOD3) gene structures, evolution, and expression. *Free Radic. Biol. Med.* 33, 337-349. 10.1016/S0891-5849(02)00905-X 12126755

[DMM049570C85] Zhang, X. M., Huang, Y., Zhang, K., Qu, L. H., Cong, X., Su, J. Z., Wu, L. L., Yu, G. Y. and Zhang, Y. (2018). Expression patterns of tight junction proteins in porcine major salivary glands: a comparison study with human and murine glands. *J. Anat.* 233, 167-176. 10.1111/joa.12833 29851087 PMC6036931

[DMM049570C86] Zhao, W., Goswami, P. C. and Robbins, M. E. (2004). Radiation-induced up-regulation of Mmp2 involves increased mRNA stability, redox modulation, and MAPK activation. *Radiat. Res.* 161, 418-429. 10.1667/3155 15038770

