# Supplementary material for: Short-term and bystander effects of radiation on murine submandibular glands
Source: Dis Model Mech. 2022 Nov 10;15(11):dmm049570. doi: 10.1242/dmm.049570 (PMC9683099; doi:10.1242/dmm.049570)
Supplement: Supplementary information [file dmm-15-049570-s1.pdf]

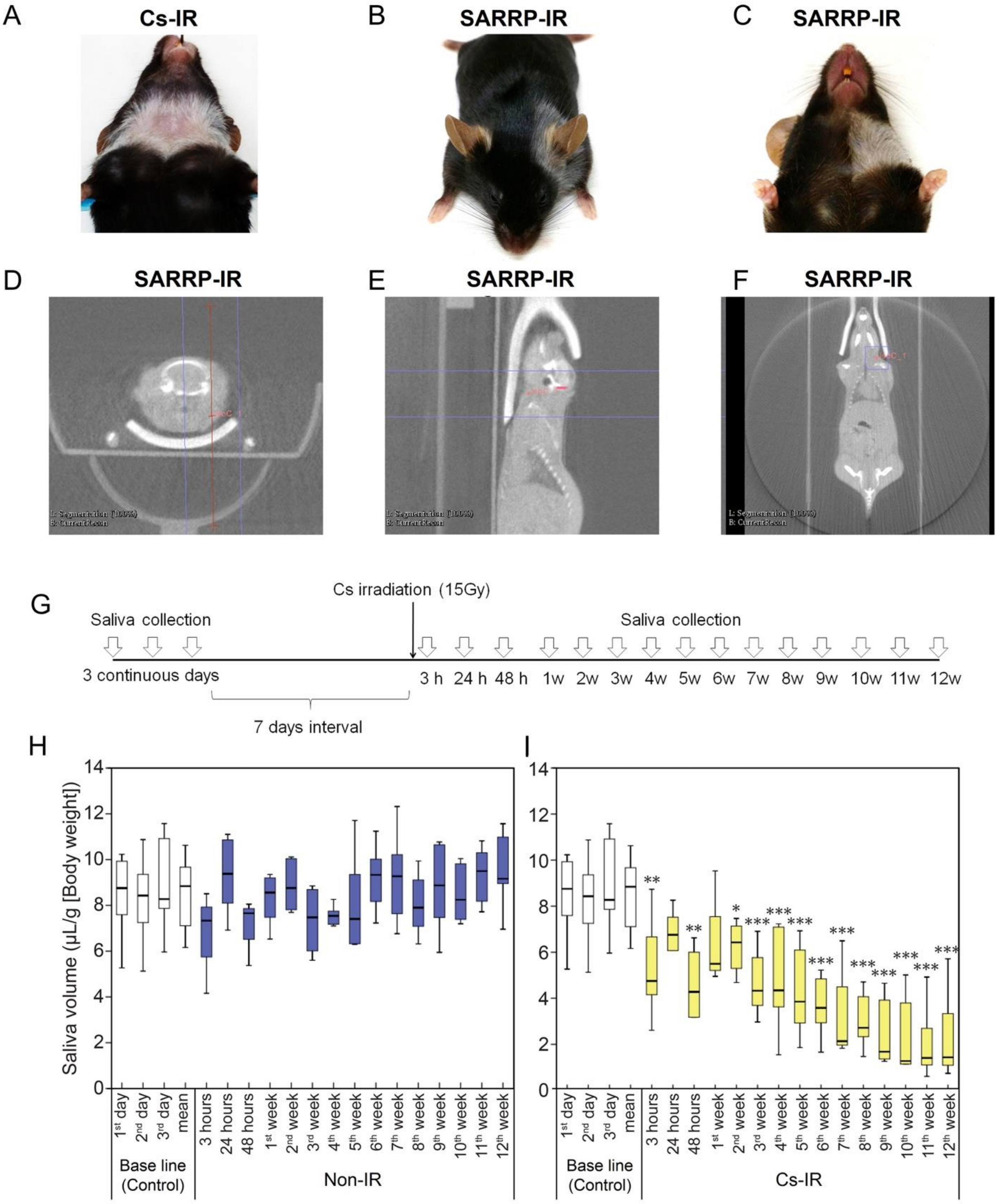

Fig. S1. A, IR using the cesium (Cs) source with a brain-slit collimator delivers a dose of 15 Gy to the entire neck region. At 3 months after IR, loss of fur color indicates the irradiated area, which includes both SMGs. **B,C,** Mice irradiated unilaterally using the small-animal radiation research platform (SARRP) show loss of fur color unilaterally, at 3 months after IR, a region that

encompasses a single SMG. **D-F,** Computed tomography (CT) images show the area targeted for IR using SARRP. Blue lines delineate area receiving radiation dose of 15 Gy. Red line and red dots indicate the isodose line and isocenter, respectively. **G,** Timeline for saliva collection from mice receiving IR from Cs source. Open arrows mark collection time points. **H, I,** Baseline saliva volume was determined for 3 days in all mice prior to IR (open boxes). Saliva volume from non-irradiated (non-IR) mice (blue boxes) and from mice irradiated (IR) using the Cs source (yellow boxes) was collected at the indicated timepoints. **H,** Saliva volumes collected from non-IR mice up to 12 weeks were not significantly different compared to baseline saliva measurements (one-way ANOVA; F = 1.246, *P*=0.251. Baseline: 1st day, n = 12 mice; 2nd and 3rd day, n = 13 mice. Non-IR controls: saliva collected at 3 hours, n = 3 mice; at 24 hours, n = 6 mice; at 48 hours, n = 3 mice; at 1st, 2nd, 4th, 5th, 7th – 12th weeks, n = 6 mice; at 3rd week, n = 4 mice; at 6th week, n = 5 mice). **I,** Saliva volume collected from IR mice decreased significantly from baseline saliva measurements over 12 weeks (one-way ANOVA; F = 14.296, *P*<0.001. IR mice: saliva collected at 3 hours, n = 5 mice; at 24 hours, n = 6 mice; at 48 hours, n = 4 mice; at 1st – 3rd, 5th – 12th weeks, n = 7mice; at 4th week; n = 6 mice). Data plotted using box-and-whisker plots. * *P*<0.05, ** *P*<0.01, *** *P*<0.001 (compared to baseline: one-way ANOVA with Dunnett's post-hoc test).

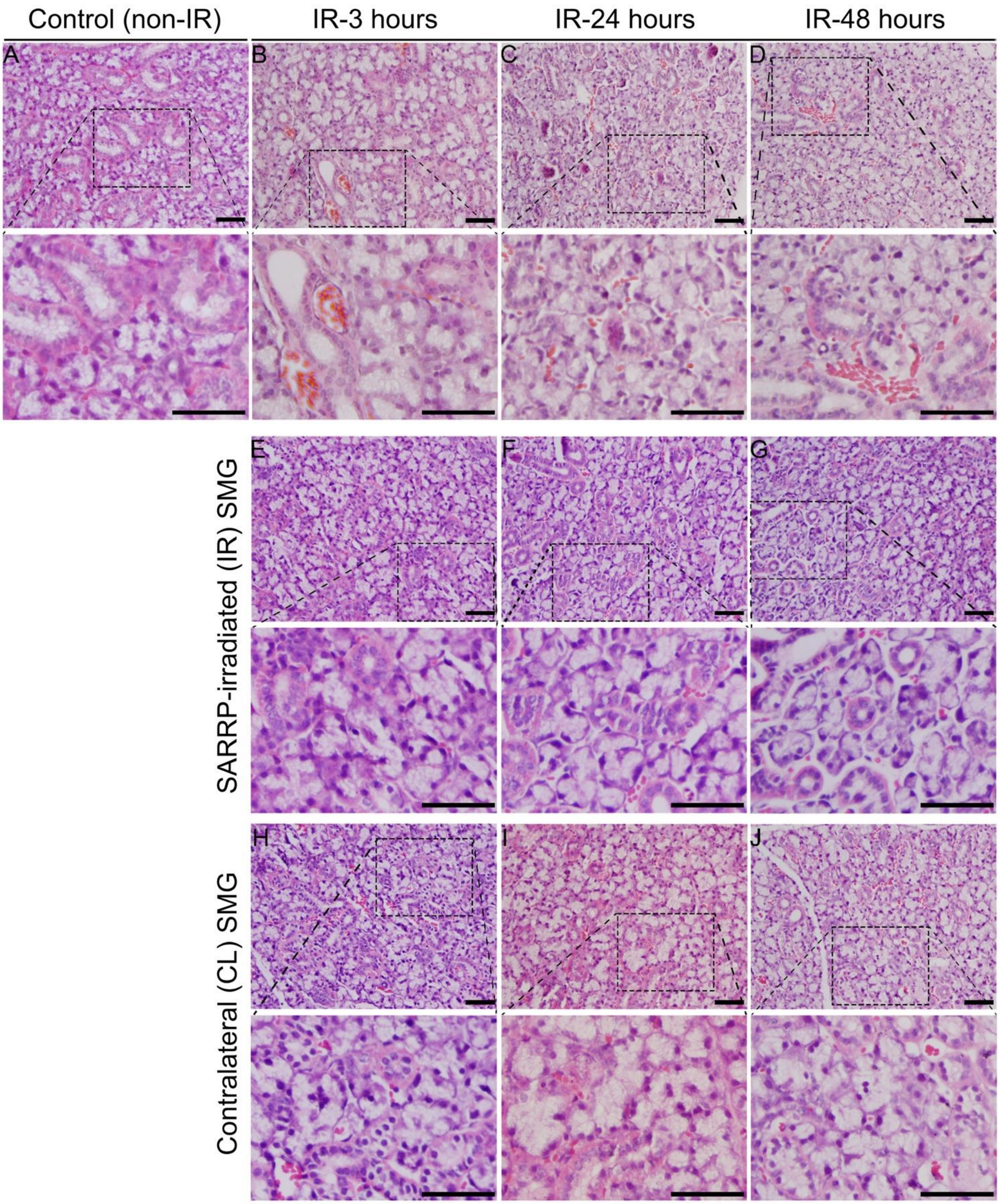
Control (non-IR)
IR-3 hours
IR-24 hours
IR-48 hours
A
B
C
D
E
F
G
H
I
J
SARRP-irradiated (IR) SMG
Contralateral (CL) SMG

Fig. S2. A-D. Hematoxylin and eosin (H&E) staining of control (**A**) and irradiated SMG sections at (**B**) 3 hours, (**C**) 24 hours and (**D**) 48 hours following IR using the Cs source. Outlined areas are shown at higher magnification in panels below. Scale bars = 50 µm. **E-G.** H&E staining of SMG unilaterally irradiated using the SARRP at (**E)** 3 hours, (**F**) 24 hours and (**G**) 48 hours following IR. Outlined areas are shown at higher magnification in panels below. **H-J.** H&E staining of contralateral (CL) SMG from mice irradiated using the SARRP at (**H**) 3 hours, (**I)** 24 hours, and (**J**) 48 hours after IR. Outlined areas are shown at higher magnification in panels below. Scale bars: 50 µm.

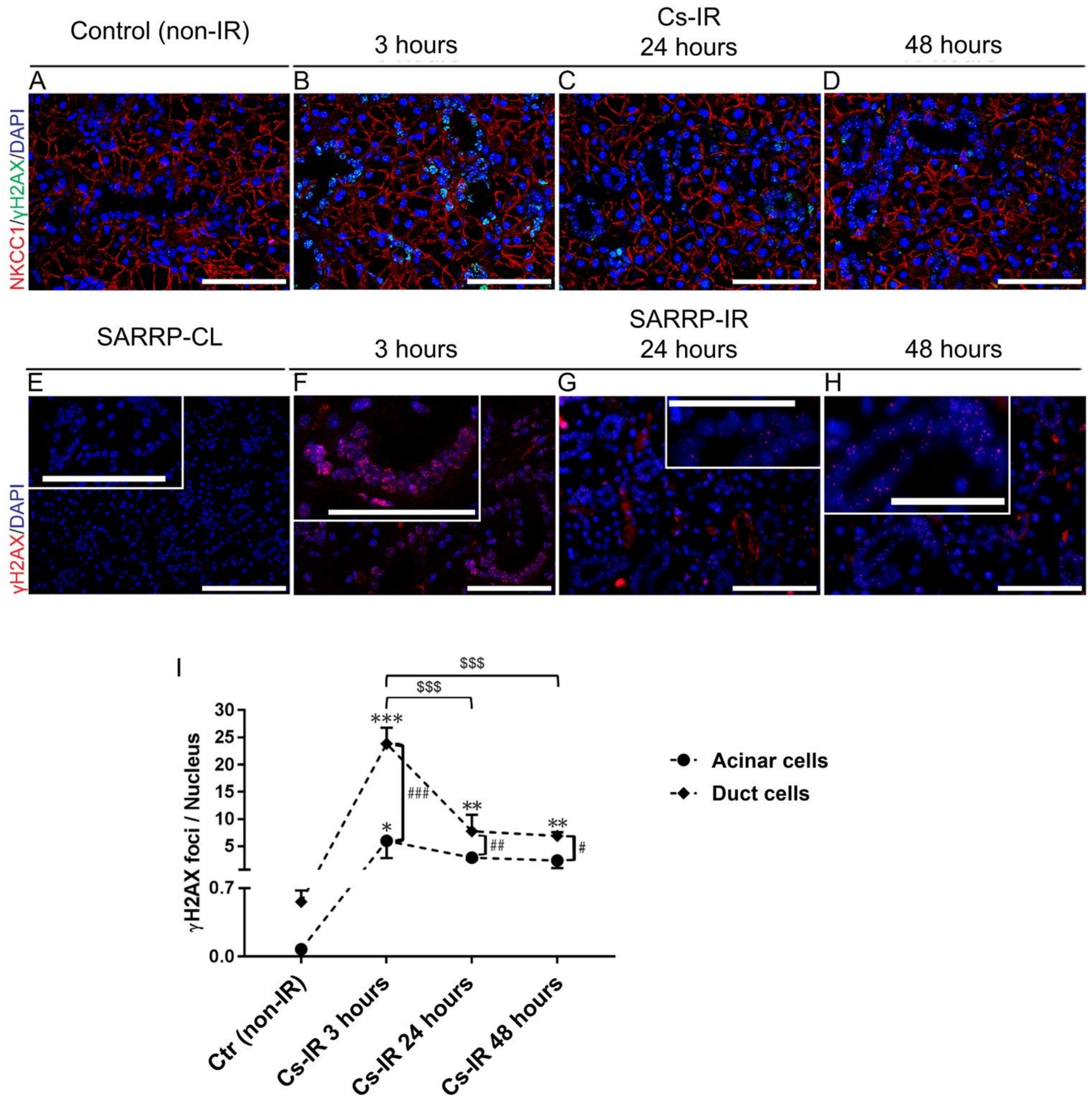

Fig. S3. A, SMGs were isolated from control and irradiated mice after IR using the Cs source at (**B**) 3 hours, (**C**) 24 hours and (**D**) 48 hours and stained with antibodies to γH2AX (green) and sodium/potassium/chloride channel NKCC1 (red). Nuclei were stained with DAPI (blue). **E,** Contralateral (CL) and irradiated SMGs were isolated from mice after unilateral IR using SARRP at (**F**) 3 hours, (**G**) 24 hours, and (**H**) 48 hours and stained with antibody to γH2AX (red). Nuclei were stained with DAPI (blue). Scale bars: 50μm. **I,** Quantification of γH2AX foci in acinar (NKCC1-

positive) cells (mean ± SD, *P*=0.011 [3 hours], *P*=0.564 [24 hours], *P*=1.000 [48 hours] vs. control) and duct (NKCC1-negative) cells (mean ± SD, *P*<0.001 [3 hours], *P*=0.002 [24 hours], *P*=0.007 [48 hours] vs. control, *P*<0.001 [24 hours], *P*<0.001 [48 hours] vs. 3 hours post-IR) (n = 3 mice, two-way ANOVA; *P*=0.765 [control], *P*<0.001 [3 hours], *P*=0.008 [24 hours], *P*=0.012 [48 hours]). Statistical analysis was performed using one-way ANOVA with Dunnett's post-hoc test (n = 3 each group): * *P*<0.05, ** *P*<0.01, *** *P*<0.001 compared to control. Statistical analysis was performed using two-way ANOVA with Bonferroni test (n = 3 each group) to compare to 3 hours post-IR ($$$ *P*<0.001) or to compare γH2AX foci in acinar and duct cells (# *P*<0.05, ## *P*<0.01, ### *P*<0.001).

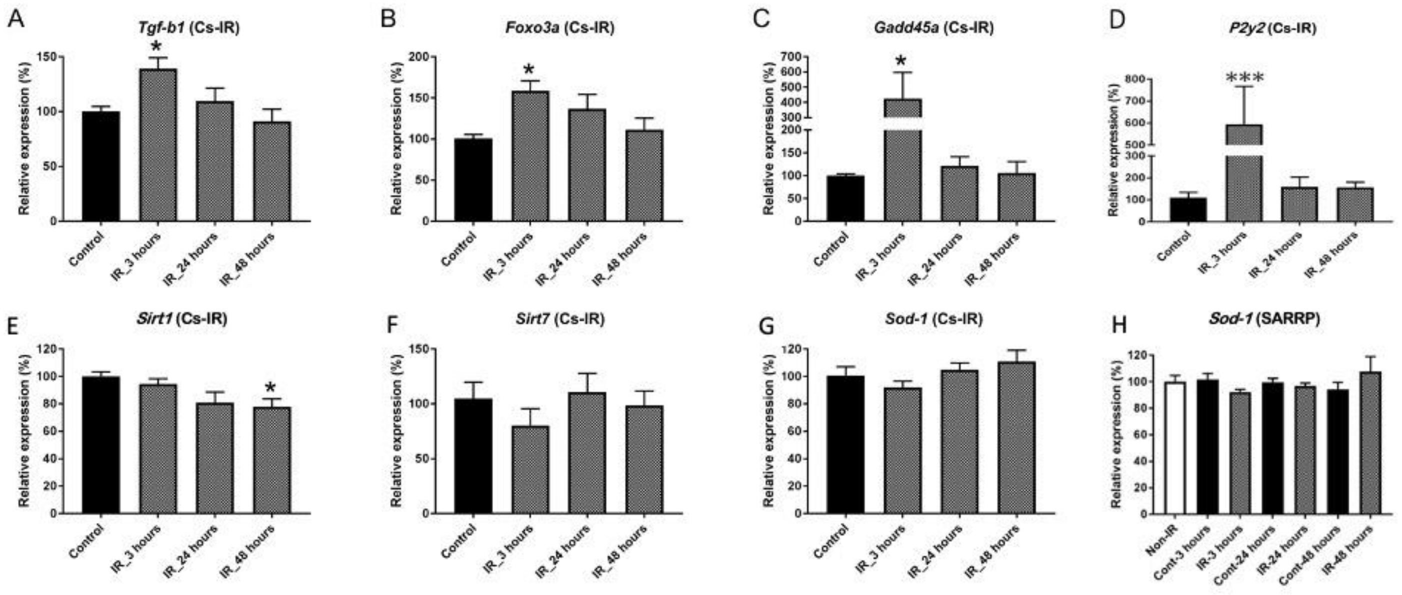

Fig. S4. A-C, Expression of (**A**) *Tgf-b1 (***P*=0.040), (**B**) *Foxo3a* (**P*=0.017) (**C**) *Gadd45a (**P=0.034) and (**D**) *P2y2* (****P*<0.001) mRNAs were transiently increased by 3 hours post-IR (Cs source) but returned to background levels by 24 hours. **E,F,** *Sirt1* and *Sirt7* levels were not changed significantly following IR (*Sirt1*: *P*=0.831 [3 hours], *P*=0.082 [24 hours], *P*=0.043 [48 hours], *Sirt7*: *P*=0.549 [3 hours], *P*=0.983 [24 hours], *P*=0.983 [48 hours]). **G,H,** Similarly, expression of *Sod-1* mRNA, which encodes a cytoplasmic protein, was not changed within 48 hours post-IR from either Cs (*Sod-1*, one-way ANOVA; F = 1.671, *P*=0.213) or SARRP source (two-way ANOVA; F=0.015, *P*=0.903). Data show mean ± SEM. Statistical analysis was performed using one-way ANOVA with Dunnett's post-hoc test and two-way ANOVA with Bonferroni test was used to compare IR and CL SMGs (n = 3-5 each group): **P*< 0.05, ****P*< 0.001 compared to control (non-IR).

Table S1. Primer sequences used for qPCR analysis.

Gene	sequence	Reference
Rps29	F AATACGGGCTGAACATGTGC<br>R AGCATGATCGGTTCCACTTG	Song et al. Commun Biol 2021
Mist1	F GCTGACCGCCACCATACTTAC<br>R TGTGTAGAGTAGCGTTGCAGG	Shubin et al. Acta Biomaterialia 2017
Aqp5	F GTGAGTGGTGGCCACATCAATCC<br>R GGGAGTCCGTGGAGGAGAAGAT	Shubin et al. Acta Biomaterialia 2017
M3r	F GGGGAACTTAGCCTGTGACC<br>R GTTGTTCGTTTGGCTCGG	Song et al. Commun Biol 2021
P2y2	F CGCTTCAACGAGGACTTCA<br>R GGTTTTGAGGCGGCATAGGA	Song et al. Commun Biol 2021
Il-1β	F GCAACTGTTCCTGAACTCAACT<br>R ATCTTTTGGGGTCCGTCAACT	Sanchez-Lopez et al. Oncogene 2016
Tnf-α	F CTATGGCCCAGACCCTCACACTC<br>R GCTGGCACCACTAGTTGGTTGTCTT	Shin et al. Cell Rep 2013
Cxcl-2	F CGCTGTCAATGCCTGAAGAC<br>R ACACTCAAGCTCTGGATGTTCTTG	Silva et al. Microb Pathog 2012
Mmp2	F CTGCAGGGTGGTGGTCATAG<br>R GCCCAGCCAGTCTGATTTGA	Hou et al. J Cell Physiol 2019
Mmp9	F CCTCCAACCTCACGGACAC<br>R TTGGAATCGACCCACGTCTG	Hou et al. J Cell Physiol 2019
Bax	F CAATATGGAGCTGCAGAGGATG<br>R CTGATCAGCTCGGGCACTTTA	Brown et al. Cell Rep 2013
Bcl-2	F CTCGTCGCTACCGTCGTGACTTCG<br>R CAGATGCCGGTTCAGGTACTCAGTC	Wu et al. Stroke 2003
Bcl-xl	F TGGAGTAAACTGGGGTCGCATC<br>R AGCCACAGTCATGCCCGTCAGG	Maksimow et al. Bool 2003
p21	F AGGCAGACCAGCCTGACAGAT<br>R TCCTGACCCACAGCAGAAGAG	Marmary et al. Cancer Res 2016
Sir1	F CAGTGTCATGGTTCCTTTGC<br>R CACCGAGGAACTACCTGAT	Rodgers et al. Nature 2005
Sirt3	F ACAGCTACATGCACGGTCTG<br>R ACACAATGTCGGGTTTCACA	Brown et al. Cell Rep 2013
Sirt7	F GCACTTGGTTGTCTACACGG<br>R CTTAGGTCGGCAGCACTCAC	Mohrin et al. Aging Cell 2018
Foxo3a	F GCAAACCCTCTCGGACTCTC<br>R TTCCCCACATTCAAACCAAC	Li et al. Eur J Pharmacol 2019
Gadd45a	F GCTGCCAAGCTGCTCAAC<br>R TCGTCGTCTTCGTCAGCA	Kaufmann et al. Gene Exp Patt 2011

Tgf-β1	F CCCGAAGCGGACTACTATGC<br>R CATAGATGGCGTTGTTGCGG	Spiegelberg et al. Mol Med 2014
Sod-1	F TGGGCAAAGGTGGAAATG<br>R AATCCCAATCACTCCACAGG	PrimerBLASTdesign with MIQE validation
Sod-2	F CATTCTGGGAAGCCATTCAG<br>R GCCGCTACTGAGAAAGGTG	PrimerBLASTdesign with MIQE validation
Pgc-1α	F AGCCGTGACCACTGACAACGAG<br>R GCTGCATGGTTCTGAGTGCTAAG	Barroso et al. Eur J Nutr 2018
Pgc-1β	F GGCAGGTTCAACCCCGA<br>R CTTGCTAACATCACAGAGGATATCTTG	Barroso et al. Eur J Nutr 2018
Zo-1	F GGGAGGGTCAAATGAAGACA<br>R GGCATTCCTGCTGGTTACAT	Ruan et al. JSC 2014

Disease Models & Mechanisms • Supplementary information

References for Supplementary Information

Barroso, W. A., Victorino, V. J., Jeremias, I. C., Petroni, R. C., Ariga, S. K. K., Salles, T. A., Barbeiro, D. F., de Lima, T. M. and de Souza, H. P. (2018). High-fat diet inhibits PGC-1α suppressive effect on NFκB signaling in hepatocytes. *Eur J Nutr* **57**, 1891-1900.doi:10.1007/s00394-017-1472-5

Brown, K., Xie, S., Qiu, X., Mohrin, M., Shin, J., Liu, Y., Zhang, D., Scadden, D. T. and Chen, D. (2013). SIRT3 reverses aging-associated degeneration. *Cell Rep* **3**, 319-27.doi:10.1016/j.celrep.2013.01.005

da Silva, J. B., Carvalho, E., Covarrubias, A. E., Ching, A. T., Mattaraia, V. G., Paiva, D., de Franco, M., Fávaro, R. D., Pereira, M. M., Vasconcellos, S. et al. (2012). Induction of TNF-alfa and CXCL-2 mRNAs in different organs of mice infected with pathogenic Leptospira. *Microb Pathog* **52**, 206-16.doi:10.1016/j.micpath.2012.01.002

Hou, X., Yang, S., Zhen Y. (2019). Licochalcone A attenuates abdominal aortic aneurysm induced by angiotensin II via regulating the miR-181b/SIRT1/HO-1 signaling. *J Cell Physiol* **234**, 7560-7568.doi:10.1002/jcp.27517

Kaufmann, L. T., Gierl, M. S. and Niehrs, C. (2011). Gadd45a, Gadd45b and Gadd45g expression during mouse embryonic development. *Gene Expr Patterns* **11**, 465-70.doi:10.1016/j.gep.2011.07.005

Li, H., Shen, L., Lv, T., Wang, R., Zhang, N., Peng, H. and Diao, W. (2019). Salidroside attenuates dextran sulfate sodium-induced colitis in mice via SIRT1/FoxOs signaling pathway. *Eur J Pharmacol* **861**, 172591.doi:10.1016/j.ejphar.2019.172591

Maksimow, M., Santanen, M., Jalkanen, S. and Hänninen, A. (2003). Responding naive T cells differ in their sensitivity to Fas engagement: early death of many T cells is compensated by costimulation of surviving T cells. *Blood* **101**, 4022-8.doi:10.1182/blood-2002-06-1904

Mohrin, M., Widjaja, A., Liu, Y., Luo, H. and Chen, D. (2018). The mitochondrial unfolded protein response is activated upon hematopoietic stem cell exit from quiescence. *Aging Cell* **17**, e12756.doi:10.1111/acel.12756

Rodgers, J. T., Lerin, C., Haas, W., Gygi, S. P., Spiegelman, B. M. and Puigserver, P. (2005). Nutrient control of glucose homeostasis through a complex of PGC-1alpha and SIRT1. *Nature* **434**, 113-8.doi:10.1038/nature03354

Ruan, Y. C., Wang, Y., Da Silva, N., Kim, B., Diao, R. Y., Hill, E., Brown, D., Chan, H. C. and Breton, S. (2014). CFTR interacts with ZO-1 to regulate tight junction assembly and epithelial differentiation through the ZONAB pathway. *J Cell Sci* **127**, 4396-408.doi:10.1242/jcs.148098

Sanchez-Lopez, E., Flashner-Abramson, E., Shalapour, S., Zhong, Z., Taniguchi, K., Levitzki, A. and Karin, M. (2016). Targeting colorectal cancer via its microenvironment by inhibiting IGF-1 receptor-insulin receptor substrate and STAT3 signaling. *Oncogene* **35**, 2634-44.doi:10.1038/onc.2015.326

Shin, J., He, M., Liu, Y., Paredes, S., Villanova, L., Brown, K., Qiu, X., Nabavi, N., Mohrin, M., Wojnoonski, K. et al. (2013). SIRT7 represses Myc activity to suppress ER stress and prevent fatty liver disease. *Cell Rep* **5**, 654-665.doi:10.1016/j.celrep.2013.10.007

Shubin, A. D., Felong, T. J., Schutrum, B. E., Joe, D. S. L., Ovitt, C. E. and Benoit, D. S. W. (2017). Encapsulation of primary salivary gland cells in enzymatically degradable poly(ethylene glycol) hydrogels promotes acinar cell characteristics. *Acta Biomater* **50**, 437-449.doi:10.1016/j.actbio.2016.12.049

Spiegelberg, L., Swagemakers, S. M., Van Ijcken, W. F., Oole, E., Wolvius, E. B., Essers, J. and Braks, J. A. (2014). Gene expression analysis reveals inhibition of radiation-induced TGFβ-signaling by hyperbaric oxygen therapy in mouse salivary glands. *Mol Med* **20**, 257-69.doi:10.2119/molmed.2014.00003

Song, Y., Uchida, H., Sharipol, A., Piraino, L., Mereness J. A., Ingalls. M. H., Rebhahn J., Newlands. S. D., DeLouise L. A., Ovitt. C. E. et al. (2021). Development of a functional salivary gland tissue chip with potential for high-content drug screening. *Commun Biol* **4**, 361.doi:10.1038/s42003-021-01876-x

Wu, C., Fujihara, H., Yao, J., Qi, S., Li, H., Shimoji, K. and Baba, H. (2003). Different expression patterns of Bcl-2, Bcl-xl, and Bax proteins after sublethal forebrain ischemia in C57Black/Crj6 mouse striatum. *Stroke* **34**, 1803-8.doi:10.1161/01.str.0000077255.15597.69
